# *Diaporthe betae* sp. nov., a new species associating with sugar beet root rot in Heilongjiang Province, China

**DOI:** 10.3389/fmicb.2025.1453460

**Published:** 2025-02-26

**Authors:** Hongtao Shao, Chunquan Ma, Bing Yu, Sixue Chen, Haiying Li

**Affiliations:** ^1^Engineering Research Center of Agricultural Microbiology Technology, Ministry of Education & Heilongjiang Provincial Key Laboratory of Ecological Restoration and Resource Utilization for Cold Region & School of Life Sciences, Heilongjiang University, Harbin, China; ^2^Heilongjiang Provincial Key Laboratory of Plant Genetic Engineering and Biological Fermentation Engineering for Cold Region & Key Laboratory of Molecular Biology, College of Heilongjiang Province, Heilongjiang University, Harbin, China; ^3^College of Advanced Agriculture and Ecological Environment, Heilongjiang University, Harbin, China; ^4^Department of Biology, The University of Mississippi, Oxford, MS, United States

**Keywords:** new species, *Diaporthe*, *Phomopsis*, new pathogen, sugar beet

## Abstract

**Introduction:**

Sugar beet (*Beta vulgaris* L.) is an economically important crop grown worldwide, but its production is threatened by root rot diseases caused by soil-borne fungi. This study aimed to identify and characterize a new pathogen causing root rot in sugar beet in Heilongjiang Province, China.

**Methods:**

During 2019 and 2023, isolates were obtained from infected sugar beet roots showing symptoms of red-brown or black lesions and tissue necrosis. The pathogenicity of the causal organism was confirmed by Koch’s postulates. The isolates were characterized based on morphological features and multilocus phylogenetic analyses. To evaluate potential control measures for this new pathogen in field conditions, the fungicides pyraclostrobin, boscalid, and fluconazole were tested for their efficacy in inhibiting the growth of this new pathogen *in vitro*.

**Results:**

The newly discovered pathogen was found to differ from previously described taxa in conidial morphology, molecular features, and disease index. Thus, the pathogen was determined to be a new species, which we called *Diaporthe betae* sp. nov. All three fungicides demonstrated significant inhibitory effects, with fluconazole showing the strongest activity and pyraclostrobin the second-highest efficacy.

**Discussion:**

The discovery of this new pathogenic fungus will help researchers elucidate the pathogenesis of sugar beet root rot and provide a theoretical basis for performing targeted monitoring, preventing diseases and implementing control measures.

## 1 Introduction

Sugar beet (*Beta vulgaris* L.) is an important crop that plays a significant role in the global sugar industry, animal feed and biofuel production (Mall et al., 2021). As a primary source of sucrose, sugar beet plays a crucial role in the global sugar industry, especially in regions with cooler climates. Due to its importance, sugar beet is grown extensively in various regions, including northern China, Europe, and North America. However, the crop faces several challenges, one of the most detrimental being root rot diseases, which are caused by a variety of soil-borne fungal pathogens. These diseases are widespread across sugar beet-growing areas worldwide, including northern China (Wolfgang et al., 2023). When suffering from soil-borne fungal infections, sugar beet roots exhibit brown–reddish-black lesions on the taproot and hypocotyl, necrosis and vascular discolouration, or severe decay (Windels, 2000). Sugar beet root rot significantly decreased the yield, juice quality, and sugar content of the products, all of which are critical to the profitability of sugar beet farming (Stevanato et al., 2019). To date, many species of soil-borne fungi, such as *Aphanomyces cochlioides*, *Calonectria montana*, *Fusarium oxysporum* f.sp. *radicis-betae*, *Macrophomina phaeseolina*, *Pythium aphanidermatum*, *Phoma betae*, *Phytophthora drechsleri*, *Rhizoctonia solani* (AG 2–2 IIIB and AG 2–2 IV), *Rhizopus crocorum*, *R. stolonifer*, and *Sclerotium rolfsii*, have been reported to cause root rot in sugar beet (Harveson, 2006; Farhaoui et al., 2023). These pathogens contribute to the disease complexity, making effective management particularly challenging. These fungi not only impair the root system, but also promote crop deterioration in the field, leading to substantial economic loss for farmers and agricultural industry.

The genus *Diaporthe* (syn. *Phomopsis*) is classified in the order Diaporthales and is one of the largest genera within the family Diaporthaceae (Senanayake et al., 2017). *Diaporthe* spp. are commonly found in various habitats, including soil, plant debris, and woody substrates, and exist as saprobes, endophytes, or plant pathogens (Dissanayake et al., 2017). As plant pathogens, *Diaporthe* spp. are known for their ability to cause diseases in a wide range of plant species, including agricultural crops, ornamental plants, and forest trees. They can infect different parts of the plant, such as leaves, stems, fruits, and seeds. *Diaporthe* species are responsible for causing diseases such as cankers, leaf spots, blights, melanoses, stem-end rot, and gummosis (Thompson et al., 2011; Hilário et al., 2020; Wrona et al., 2020; Caio et al., 2021; Jiang et al., 2021). The taxonomy and identification of *Diaporthe* species can be challenging due to their morphological similarities and the presence of cryptic species (Lawrence et al., 2015). The identification of *Diaporthe* species typically involves a polyphasic approach that involves examining morphological traits, performing phylogenetic analyses using multiple genetic loci, and conducting phytopathological analyses (Yashuang et al., 2020; Norphanphoun et al., 2022).

In 2021, we reported that *Ca. montana* can cause root rot in sugar beet (Shao and Li, 2021). The result indicates that sugar beet may harbor new pathogens that have not been reported. Surveys were conducted to identify the potential sugar beet root rot pathogens in sugar beet growing areas in northern China. We obtained 256 purified isolates, among which seven isolates were found to belong to the genus *Diaporthe* spp. Examinations of morphological features and multilocus phylogenetic analyses revealed that one species was different from previously described taxa and thus a new species.

## 2 Materials and methods

### 2.1 Collection of plant samples

A total of 224 diseased sugar beet roots were collected from 10 sites in sugar beet-producing regions in northern China, including Heilongjiang Province and the Inner Mongolia Autonomous Region, between 2019 and 2023. Plants showing root rot symptoms were uprooted, and the samples were carried to the laboratory for culturable fungi isolation as quickly as possible. The total number of samples collected is given in Table 1.

**Table 1:** Root samples collected from 10 sampling sites in the sugar beet growing regions in the north of China including Heilongjiang Province and the Inner Mongolia Autonomous Region during 2019 and 2023.

Region/Province	Site	Number of root samples
the Inner Mongolia Autonomous Region (China)	Qianqi	10
	Zhongqi	10
	Zhaqi	10
	Taonan	10
	Tuquan	10
Heilongjiang Province (China)	Shuangyashan	15
	Yian	15
	Nongkeyuan^a^	15
	Tianyan^b^	94
	Guoyuancun	35
Total for all sites		224

^aHarbin Academy of Agricultural Sciences innovation base. bChinese Academy of Agricultural Sciences Sugar Beet Research Institute experimental site.^

### 2.2 Isolation of culturable fungi from the root tissues

Root samples were first rinsed with a water gun to remove surface soil, blotted with sterilized paper to remove surface water, photographed, and relevant information were recorded. For each root sample, the surfaces of three pieces of root tissue, which contained both healthy and infected parts, were sterilized. The sterilization process involved immersing the samples in 1% sodium hypochlorite for one minute, followed by a fifteen-second immersion in 75% ethanol. The samples were then rinsed twice with sterilized distilled water (Shao and Li, 2021). The samples were placed on sterilized paper for drying, plated on water agar (WA) added with penicillin G (50 mg/L), and cultured in the dark. The Petri dishes were inspected daily, and any outgrowing mycelia were subcultured immediately. Isolates were purified using a single hyphal tip and preserved in potato dextrose agar (PDA) slants at 4°C. All purified cultures were deposited at the Heilongjiang University Microbiological Culture Collection Centre (HUMCC), Harbin, China. The specimens of the new identified species from this study were deposited in the Herbarium of Microbiology and Phytopathology, Heilongjiang University (HMPHU). Taxonomic information for the new taxa was submitted to Fungal Name [Fungal Names (nmdc.cn)].

### 2.3 DNA extraction, PCR, and sequencing

Purified cultures were grown on PDA for one week at 25°C, after which actively growing mycelia were transferred to 1.5 mL Eppendorf tubes. Total genomic DNA was extracted using the CTAB method (van Burik et al., 1998). The extracted DNA was dissolved in 30 μL ddH_2_O. To confirm the species, we sequenced the internal transcribed spacer (ITS) regions of all the isolates. Once the isolates were identified as belonging to the genus *Diaporthe*, amplification of four other loci, including calmodulin (*CAL*), histone H3 (*HIS*), translation elongation factor 1-alpha (*TEF1*), and *β*-tubulin (*TUB2*) genes, was performed. The primer pairs used in this study are listed in Supplementary Table S1. The PCR mixture used to amplify the different loci consisted of 13.5 μL of 2 × Es Taq Mixer (Dye) (Cwbio Group, China), 1 μL of forward primer, 10 μM (Sangon Biotech, Shanghai, China), 1 μL of reverse primer, 10 μM (Sangon Biotech, Shanghai, China), and 2 μL (100 ng/μL) of the DNA samples was added as the template for each PCR. The amplifications were carried out in 35 μL reaction volumes on a Veriti® 96-Well thermal cycler. The PCR conditions consisted of an initial denaturation step at 95°C for 5 min, followed by 35 cycles of denaturation at 95°C for 30 s, annealing at specific temperatures (57°C for ITS, 56°C for *CAL*, 63°C for *HIS*, 48°C for *TEF1*, and 58°C for *TUB2*) for 30 s, extension at 72°C for 30 s, and a final elongation step at 72°C for 7 min. The PCR amplicons were purified and sent to Sangon Biotech (Changchun, China) for sequencing. The resulting sequences were assembled using SeqMan from DNASTAR’s Lasergene v. 7 software. All sequences obtained from the isolates in this study were submitted to GenBank (accession numbers: MW882216 to MW882224, PP064117 to PP178567) (Supplementary Table S2).

### 2.4 Phylogenetic analyses

The ITS region, calmodulin (*CAL*), histone H3 (*HIS*), translation elongation factor 1-alpha (*TEF1*), and *β*-tubulin (*TUB2*) gene sequences were subjected to comparison with the GenBank database using BLASTn for species identification and selection of closely related strains displaying high homology. To enhance the accuracy of the phylogenetic tree constructed, other strains of *Diaporthe* with low homology were also selected for the following procedure (Supplementary Table S2). The sequences of each region/gene were aligned using the online service MAFFT version 7.525 with iterative refinement methods (FFT-NS-i)^1^ (Katoh et al., 2019). Afterward, TrimAl accessible through PhyloSuite (Zhang et al., 2020) was utilized to trim ambiguously aligned positions or regions (Capella-Gutiérrez et al., 2009). To generate concatenated sequences, the different single gene sequences were assembled using PhyloSuite software (Zhang et al., 2020). Corresponding to the concatenated five loci, the ITS sequences covered positions 1–578, *CAL* sequences covered positions 579–896, *HIS* sequences covered positions 897–1,321, *TEF1* sequences covered positions 1,322–1,697, and *TUB2* sequences covered positions 1,698–2,473. DAMBE (Data Analysis in Molecular Biology and Evolution) was employed to assess substitution saturation in the concatenated sequences before phylogenetic analysis (Xia and Xie, 2001). Phylogenetic and molecular evolutionary analyses of sequence data of four isolates from this study and those of 187 isolates used in previous study were listed in Supplementary Table S2. Bayesian inference (BI) analysis of the combined sequence datasets (ITS + *CAL* + *HIS* + *TEF1* + *TUB2*, 2,473 bp) was conducted using MrBayes v3.2.7a (Ronquist et al., 2012) accessible through PhyloSuite (Zhang et al., 2020), with the best nucleotide substitution models selected based on Bayesian information criterion (BIC) using ModelFinder v. 1.6.8 with the greedy algorithm (Kalyaanamoorthy et al., 2017; Supplementary Table S3). Markov Chain Monte Carlo (MCMC) simulations were run. Sampling was conducted every 100 generations until the average standard deviation of split frequencies fell below 0.01, at which point the simulations were terminated. Trees were summarized after discarding 250 burn-in samples, and posterior probabilities (PP) were computed for each branch. Maximum likelihood phylogenies on the five concatenated gene datasets (ITS + *CAL* + *HIS* + *TEF1* + *TUB2*, 2,473 bp) were inferred using the IQ-TREE v 1.6.8 (Nguyen et al., 2015) under Edge-linked partition model accessible through PhyloSuite (Zhang et al., 2020). Branch support was evaluated using a bootstrap analysis and Shimodaira-Hasegawa-like approximate likelihood ratio test (SH-aLRT) with 1,000 replicates (Guindon et al., 2010).

### 2.5 Identification of the *Diaporthe* pathogens

The isolates were cultured in 2% WA media supplemented with healthy autoclaved soybean (*Glycine max*) stems (30 min, 121°C, 1.2 bar, autoclaved twice). The plates were maintained under a black light blue fluorescent lamp (BLB) at 25°C for more than one month to induce the formation of fruiting bodies (Ohsawa and Kobayashi, 1989). At least 30 conidiomata, alpha and gamma conidia were measured to calculate the mean size and standard deviation (SD). The colony morphology was assessed by observing cultures grown on PDA for 10 days at 25°C, following a 12-h light/dark cycle. The micromorphological features were examined and documented using a Nikon SMZ1270 stereomicroscope equipped with a 20 M SCCD and an Olympus BX53 microscope equipped with an Olympus DP25 colour digital camera for capturing images of the fungal structures.

### 2.6 Pathogenicity of the *Diaporthe* pathogens

Two isolates (HHL19101101S2D1 and 23092201B), which were isolated from different regions and in different years, were selected for pathogenicity testing. Each pot was filled with a sterile mixture of soil, sand, and vermiculite (in a 1:1:1 ratio, v:v:v) that had been air-dried at 160°C for five hours, with eight disinfected seeds sown in each pot. The pots were organized in a greenhouse using a randomized block design, with night/day temperatures set at 20/25°C. Three weeks after emergence, the treatment pots were inoculated with eight discs (5 mm diameter) of agar with actively growing mycelium from a 7-day-old colony. The control pots, on the other hand, were inoculated with eight discs (5 mm diameter) of 2% WA. Each isolate was tested using five replicate pots. Two weeks after inoculation, the remaining plants and the dead plants were collected and rinsed. Disease severity was evaluated using the following scale: 0, no disease; 1, one-fourth of the root was necrotic; 2, one-half of the root was necrotic; 3, three-fourths of the root was necrotic; and 4, the whole root was necrotic, or the seedling were completely dead (Supplementary Figure S1). The experiments were repeated twice, and the scales were converted to a disease severity index (DSI) using the following equation:

$$DSI=\frac{\sum \left(\mathrm{Disease scale}\times \mathrm{number of plants in this scale}\right)}{\mathrm{Total number of plants tested}\times 4}\times 100$$

To verify Koch’s postulates, the inoculated fungi were reisolated by excising small portions of necrotic tissue from the edges of four lesions and culturing them on WA plates at 25°C. Species identity was confirmed based on morphology. Before conducting analysis of variance (ANOVA), the DSI data were square root-transformed. The average DSIs of the isolates were then compared using Fisher’s protected least significant difference (LSD) test at a significance level *p* < 0.05. Statistical analyses were carried out using SPSS software version 21.0 (IBM Corporation, Armonk, New York, USA).

### 2.7 Assessment of fungicides against *Diaporthe betae in vitro*

To determine potential control measures for this pathogen in the field, the fungicides pyraclostrobin, boscalid, and fluconazole were tested for their ability to inhibit *D. betae* growth *in vitro*. The three tested chemical fungicides were prepared in a 200 μg/mL stock solution according to the manufacturer’s instructions and then diluted to different concentrations. When the sterilized PDA culture medium was cooled to 50°C, 2 mL of each fungicide solution (at the final concentrations 0.1, 1, 10, 20, or 100 μg/mL) was added to 18 mL of the culture medium, and mixed thoroughly. As a control (CK), 2 mL of sterile water was added to 18 mL of culture medium. After the medium was solidified, a plug taken from the *D. betae* culture using a 7 mm punch was placed in the center of the medium. When the mycelium in the CK group covered two-thirds of the Petri dish, the radius of fungal growth on each plate was measured using the cross method and the inhibition rate was calculated (Zou et al., 2013). The experiment was repeated twice.

$$\mathrm{Inhibition Rate}\;\left(\%\right)=\frac{\left(R\mathrm{control}-R\mathrm{treatment}\right)}{\left(R\mathrm{control}-\mathrm{Agar disc diameter}\right)}\times 100\%$$

*R*control = Radius of fungal growth in the untreated CK group

*R*treatment = Radius of fungal growth in the treatment group (i.e., each of the different concentrations of pyraclostrobin, boscalid and fluconazole).

## 3 Results

### 3.1 Sampling and isolation

**Figure 1:**
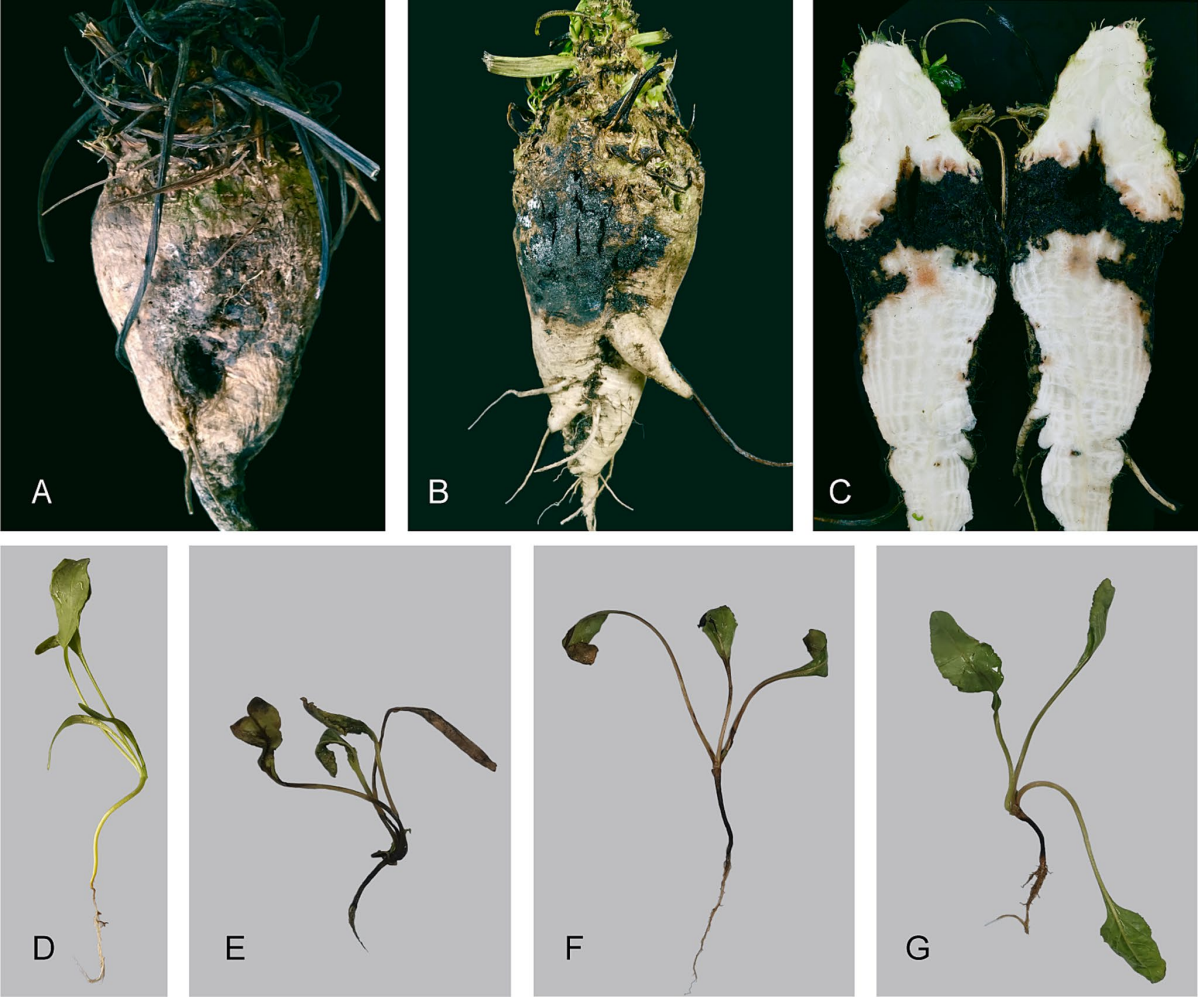
Symptoms of sugar beet root rot caused by *Diaporthe betae* sp. nov. **(A–C)** Disease symptoms of root rot caused by *Diaporthe betae* sp. nov. in the field. **(D)** Control plant; and **(E–G)** disease symptoms of root rot caused by *Diaporthe betae* sp. nov. after two weeks.

During the survey of sugar beets, roots with brown–reddish-black lesions on the taproot or hypocotyl or with necrosis/severe decay in the root tissues were gathered. Pure-culture isolation from 224 diseased root samples collected from 10 sites yielded 256 isolates, which were found to represent 12 distinct taxa based on mycelial morphology and ITS rDNA sequencing. Of these isolates, seven were identified as *Diaporthe* spp. Among these seven isolates, three were collected from the Chinese Academy of Agricultural Sciences Sugar Beet Research Institute experimental site (45°59′53.06″N, 126°38′24.16″E) in 2019, two were from the Harbin Academy of Agricultural Sciences Innovation Base (45°51′46.71″N, 126°28′21.81″E) in 2023, and two were from Guoyuan village (46°01′48.19″N, 126°39′39.87″E) in 2023. Sugar beet roots infected by *Diaporthe* exhibited brown–reddish-black, irregularly shaped lesions on the taproot. With the progression of the disease, necrosis extended from the outer layers toward the inner tissue, and the cross-sections of the inoculated sugar beet roots showed black discolouration, with internal tissue rotting (Figures 1A–C). Among the seven isolates, four had more than 99% similarity according to the BLASTN analysis (Supplementary Tables S4–S7). Phylogenetic tree analysis was performed with these isolates, while the remaining three were discarded.

### 3.2 Phylogenetic analysis

**Figure 2:**
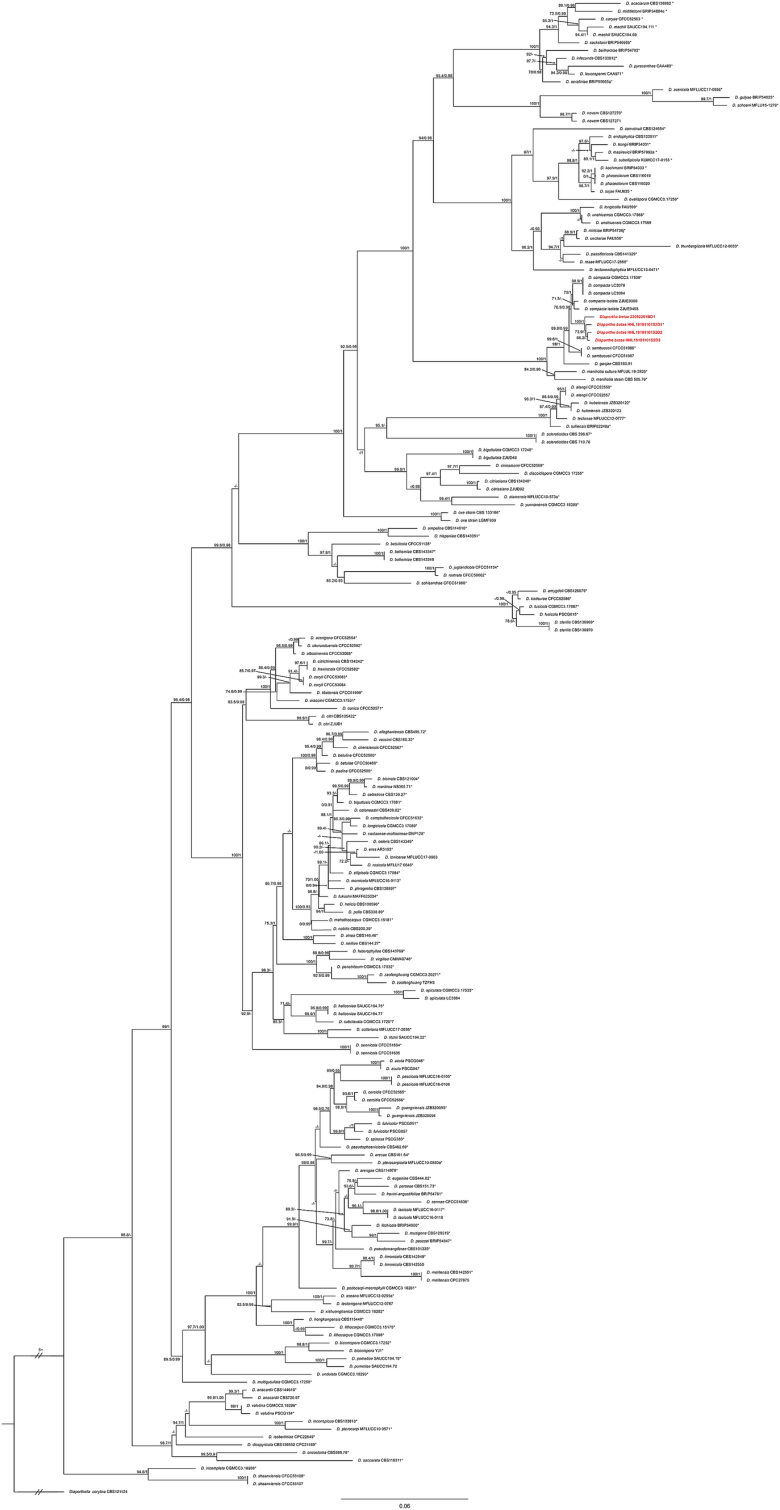
Phylogenetic tree of *Diaporthe* resulting from a maximum likelihood analysis based on the combined ITS, *CAL*, *HIS*, *TEF1*, and *TUB2* regions. The ML bootstrap support values (left) above 70% and Bayesian posterior probabilities (right) above 0.90 are indicated at the nodes. The isolates identified in this study are highlighted in red and bold. Ex-type/ex-epitype isolates are indicated by an asterisk (*). *Diaporthella corylina* (CBS 121124) was used as the outgroup taxon.

A larger possible taxa database was selected for phylogenetic analysis to improve classification accuracy. The combined dataset (ITS + *CAL* + *HIS* + *TEF1* + *TUB2*) used for phylogenetic analyses consisted of 812 sequences from 191 isolates, including 20 sequences (4 for ITS, 4 for *CAL*, 4 for *HIS*,4 for *TEF1*, and 4 for *TUB2*) newly generated in the present study. The phylogenetic analysis included a total of 2,473 characters including gaps (578 for ITS, 318 for *CAL*, 425 for *HIS*, 376 for *TEF1*, and 776 for *TUB2*). Of these characters, 1,058 characters were constant (proportion = 0.42782), 268 variable characters were parsimony-uninformative. Number of parsimony-informative characters was 1,147. The maximum likelihood (ML) analysis resulted in a best scoring IQ-TREE tree with a final optimized likelihood value of – 40779.597. Similar tree topologies were obtained by the ML and BI methods. The phylogenetic analysis showed that four *Diaporthe* isolates (HHL19101101S2D1, HHL19101101S2D2, HHL19101101S2D3, and 23092201B) formed a separate clade closely related with *D. compacta*, supported by 100% bootstrap and a Bayesian posterior probability (BPP) value of 1.0 (Figure 2). Based on the multilocus phylogeny and morphology, the four isolates from sugar beet (*Beta vulgaris* L.) roots were assigned to a newly described taxon and characterized as described below.

Taxonomy

**Figure 3:**
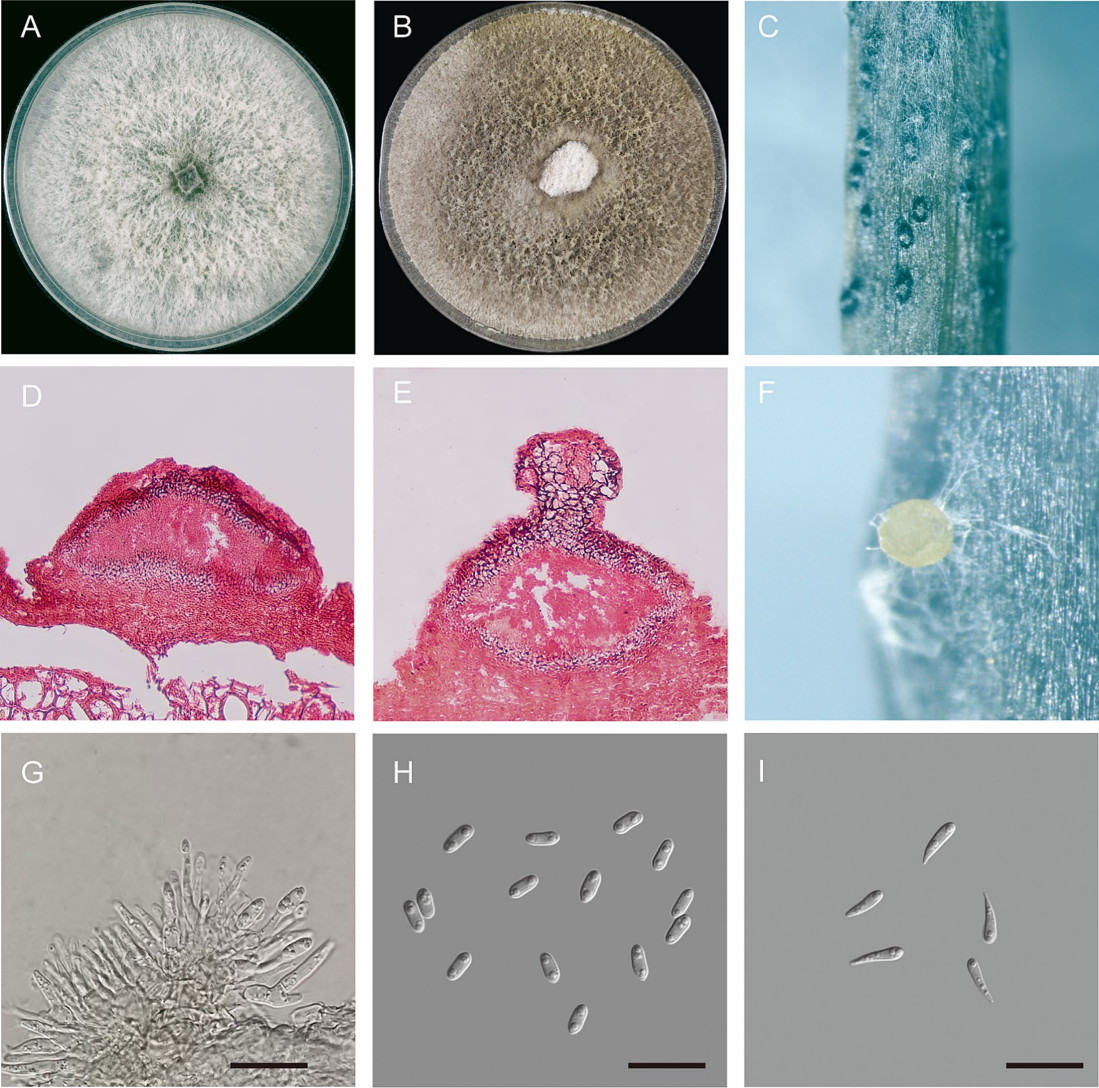
Morphological characteristics of *Diaporthe betae* sp. nov. **(A)** Seven-day-old colonies on PDA; **(B)** fifteen-day-old colonies on PDA; **(C)** conidiomata on a soybean (*Glycine max*) stem; **(D,E)** section view of conidiomata; **(F)** conidiomata with conidial droplets on a soybean (*G. max*) stem; **(G)** conidiogenous cells; **(H)** alpha-conidia; and **(I)** gamma conidia – scale bars: **(G,H,I)** = 20 μm.

*Diaporthe betae* Hongtao Shao and Haiying Li, sp. nov. (Figure 3).

Etymology: *betae*, refers to the epithet of the host plant *Beta vulgaris* L.

Type specimen: China, Heilongjiang Province, Hulan, Chinese Academy of Agricultural Sciences Sugar Beet Research Institute experimental site, on root rot of sugar beet (45°59′53.06″N, 126°38′24.16″E), 4 October 2019, H.T. Shao holotype HMPHU 3001 = HHL19101101S2D1.

Additional material: China, Heilongjiang Province, Hulan, Chinese Academy of Agricultural Sciences Sugar Beet Research Institute experimental site (45°59′53.06″N, 126°38′24.16″E), on root rot of sugar beet, 4 October 2019, H.T. Shao ex-type living culture HUMCC 3268 = HHL19101101S2D2, HUMCC 3269 = HHL19101101S2D3, China, Heilongjiang Province, Harbin, Harbin Academy of Agricultural Sciences Innovation Base (45°51′46.71″N, 126°28′21.81″E), on root rot of sugar beet, 27 January 2023, H.T. Shao, ex-type living culture HUMCC 3126 = 23092201B.

Fungal name: FN 571889.

Description: Sexual morphology was not observed. Pycnidial conidiomata were produced on soybean stems; They were solitary or aggregated, dark, subglobose in shape, coated with short hyphae, superficial or semi-immersed, and 86.7–246.41 × 122.63–388.96 μm in size (*n* = 30). When mature, milk-white conidial drops were released from the ostioles. Conidiophores were hyaline, smooth, unbranched or branched, straight or slightly curved, broader at the base and cylindrical, occurred in dense clusters, tapered toward the apex, and 6.92–18.14 × 0.9–3.42 μm (mean ± SD = 12.9 ± 2.79 × 2.35 ± 0.63, *n* = 50). Alpha conidia were abundant, unicellular, ellipsoid to cylindrical, hyaline, aseptate, obtusely rounded at both ends, bi- or multi-guttulate, and 6.5–10.2 × 2.5–4.0 μm (mean ± SD = 7.9 ± 0.6 × 3.2 ± 0.3 μm, *n* = 50). length to width (L/W) ratio = 2.47 (Table 2). Beta conidia were not observed. Gamma conidia were hyaline, aseptate, multi-guttulate, and 8.1–14.9 × 1.9–3.7 μm (*n* = 30).

**Table 2:** Length of alpha conidia, width of alpha conidia, and L/W ratios of *Diaporthe betae* and its closely related species *Diaporthe compacta* and *Diaporthe sambucusii*.

Species	isolate	L^a^	W^b^	L/W ratios	References
*Diaporthe betae*	HHL19101101S2D1	7.9	3.2	2.47	This study
*Diaporthe compacta*	CGMCC3.17536	6.6	2.5	2.64	Gao et al. (2016)
*Diaporthe sambucusii*	CFCC51986	8.5	2.2	3.86	Yang et al. (2018)

^aLength of alpha conidia. bWidth of alpha conidia.^

Culture characteristics: Colonies on PDA exhibited fluffy aerial mycelium and radial growth; were initially silvery white and turned olivaceous or dark gray on the surface and olivaceous black in the reverse; and reached 90 mm in diameter after 10 days at 25°C. Pycnidia were not observed.

Notes: Four isolates of *D. betae* formed a strongly supported clade (ML/BI = 100/1) and showed close relatedness to *D. compacta* Y.H. Gao & L. Cai (97% in *CAL*, 98–99% in *HIS*, 96–98% in *TEF1*, and 96–99% in *TUB2*). *D. betae* differs from *D. compacta* in morphology, producing larger alpha conidia (6.5–10.2 × 2.5–4.0 in *D. beta* vs. 6.0–7.5 × 2.0–3.0 μm in *D. compacta*), lacking beta conidia, and having gamma conidia.

### 3.3 Pathogenicity of the *Diaporthe* pathogens

The two *D. betae* isolates were both pathogenic, and isolate 23092201B was more aggressive than isolate HHL19101101S2D1 (Table 3). Symptoms begin as dark brown lesions beneath the soil surface and progress up the hypocotyls, often causing the hypocotyls to wrinkle (Figures 1E–G; Supplementary Figure S2). The induced symptoms were consistent with those observed in the fields. No lesions were induced in the control roots inoculated with WA (Figure 1D). *D. betae* was successfully reisolated from inoculated seedling roots and was identical to the original isolates HMPHU 3001 (HHL19101101S2D1) and HUMCC 3126 (23092201B). Thus, Koch’s postulates were met, and *D. betae* was the causal agent of the disease.

**Table 3:** Disease index of the isolates HHL19101101S2D and 23092201B (*Diaporthe betae* sp. nov.) inoculated on sugar beet seedlings.

Treatment	Disease index
23092201B	8.24^a^
HHL19101101S2D1	6.95^b^
Control	0^c^

Means in a column followed by different letters are significantly different according to Fisher’s protected least significant differences (LSD) test at *p* < 0.05.

### 3.4 Assessment of fungicides against *Diaporthe betae in vitro*

**Figure 4:**
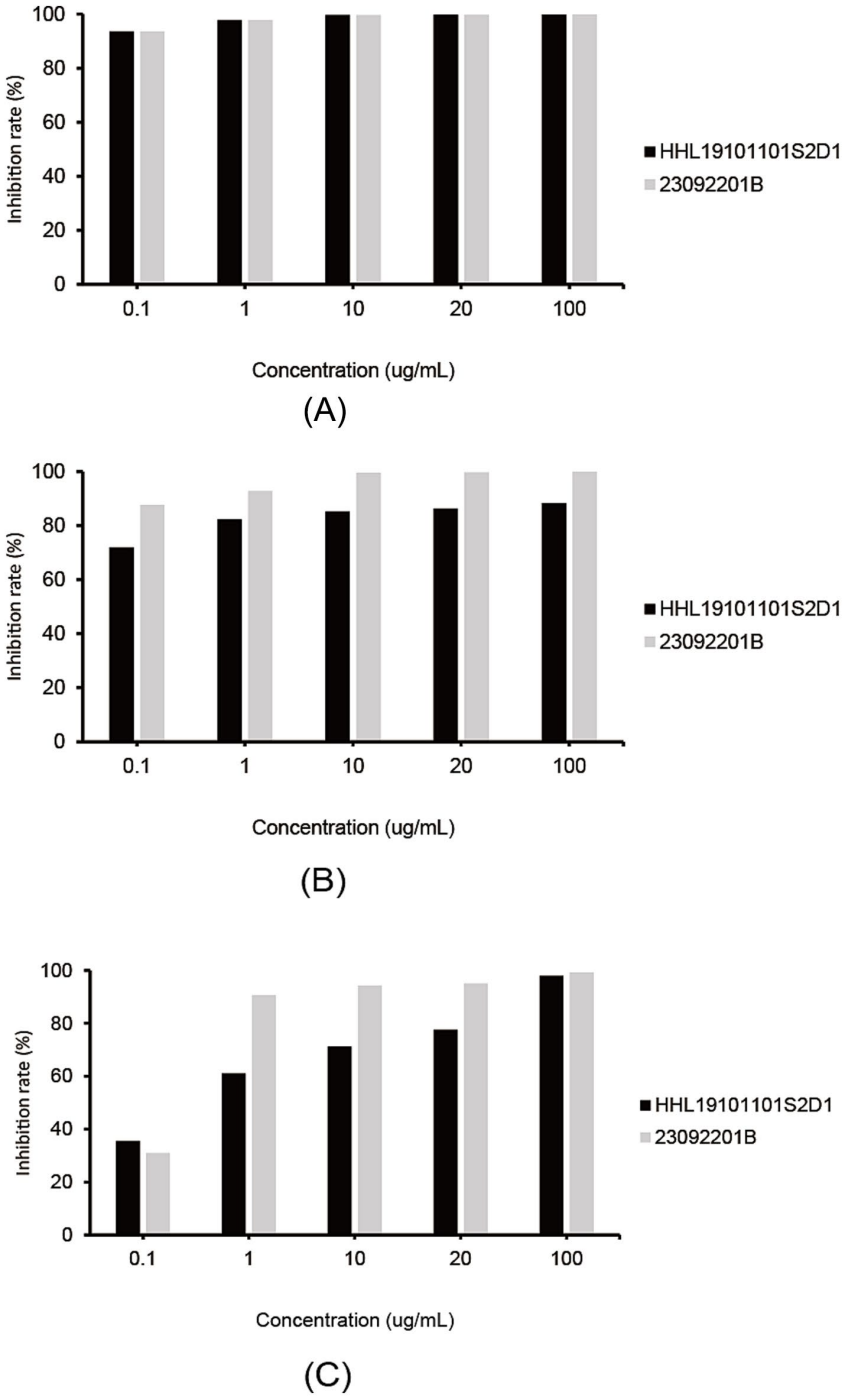
Efficacy of three fungicides in controlling *Diaporthe betae* isolates HHL19101101S2D and 23092201B on plates. **(A)** Inhibitory effect of fluconazole on *Diaporthe betae* isolates; **(B)** inhibitory effect of pyraclostrobin on *Diaporthe betae* isolates; and **(C)** inhibitory effect of boscalid on *Diaporthe betae* isolates.

All three fungicides exhibited inhibitory effects against the isolates on the plate. Fluconazole showed the overall best inhibitory effect, with mycelial growth inhibition rates of 93.7% for both isolates at 0.1 μg/mL (Figure 4A). Pyraclostrobin showed the second-best inhibitory effect on isolate HHL19101101S2D1 (86.4%) and on isolate 23092201B (~100%) at 20 μg/mL (Figure 4B). Boscalid had the lowest inhibitory effect, with inhibition rates of 77.5 and 95.1%, respectively, at 20 μg/mL (Figure 4C). Clearly, isolate 23092201B was more sensitive to boscalid and pyraclostrobin than isolate HHL19101101S2D1.

## 4 Discussion

Sugar beet root rot occurs worldwide and is caused by a broad range of fungi. Many species have been identified as causal agents of sugar beet root rot, including *Aphanomyces cochlioides*, *Calonectria montana*, *Fusarium oxysporum* f.sp. *radicis-betae*, *Macrophomina phaeseolina*, *Pythium aphanidermatum*, *Phoma betae*, *Phytophthora drechsleri*, *Rhizoctonia solani* (AG 2–2 IIIB and AG 2–2 IV), *Rhizopus crocorum*, *R. stolonifer*, and *Sclerotium rolfsii* (Harveson, 2006; Shao and Li, 2021; Farhaoui et al., 2023). Our research demonstrated the presence of a new pathogen, *Diaporthe betae* sp. nov., on sugar beet roots in Heilongjiang Province, China. Sugar beet roots infected by *Diaporthe* exhibited brown–reddish-black, irregularly shaped lesions on the taproot in the field. As the disease progresses, necrosis penetrates from the external layers into the inner tissue. *Phoma betae* (syn. *Neocamarosporium betae*, *Pleospora betae*), the causal agent of *Phoma* root rot, has been documented in sugar beet cultivation regions across Australia, Asia, Europe, and North America (Farhaoui et al., 2023). In the field, *P. betae* can induce seedling damping-off, root rot, and leaf spot, while also contributing to decay in storage piles. Through the utilization of NCBI BLAST and phylogenetic analysis, it was unequivocally shown that the newly discovered pathogen belongs to the genus *Diaporthe*. Phylogenetically, *Phoma* is classified to Pleosporales, Dothideomycetes (Deb et al., 2020), while *Diaporthe* belongs to Diaporthales, Sordariomycetes. In addition, morphological analyses revealed that the size and shape of the alpha conidia of *D. betae* were different from those of *P. betae* (Supplementary Figure S3). Phylogenetic analyses revealed that *D. betae* formed a well-separated clade with statistical support (ML/BI = 100/1), and showed close relatedness to *D. compacta* (97% in *CAL*, 98–99% in *HIS*, 96–97% in *TEF1*, and 96–99% in *TUB2*) and *D*. *sambucusii* (99% in *CAL*, 97% in *HIS*, 97–98% in *TEF1*, and 97–98% in *TUB2*). Efforts to stimulate the creation of sexual forms of *D. betae* were unsuccessful. Only the asexual forms of *D. betae* are generated when grown on soybean stems, whereas when cultured on other media containing pine needles, or typical fungal media, no asexual forms are produced. On the other hand, the asexual forms of *D. compacta* can be triggered in pine needle medium (PNP) (Gao et al., 2016), and the asexual forms of *D*. *sambucusii* can be cultivated on PDA (Yang et al., 2018). *D. betae* differs from *D. compacta* in morphology, producing larger alpha conidia (6.5–10.2 × 2.5–4.0 in *D. beta* vs. 6.0–7.5 × 2.0–3.0 µm in *D. compacta*), lacking beta conidia, and having gamma conidia (Gao et al., 2016). *D. betae* differs from *D*. *sambucusii* in producing larger alpha conidia (6.5–10.2 × 2.5–4.0 in *D. beta* vs. 7.0–9.5 × 2.0–2.5 μm in *D*. *sambucusii*) with obtusely rounded ends, lacking beta conidia, and having gamma conidia, while *D*. *sambucusii* having alpha conidia with acutely rounded ends, producing beta conidia, and lacking gamma conidia (Yang et al., 2018). It is noteworthy that there is the difference in the L/W ratios of the alpha conidia of *D. betae*, *D. compacta*, and *D*. *sambucusii* (Table 2). This information is valuable in distinguishing between these close related fungal species based on their conidial morphology. Given the deformability, plasticity, and overlapping of morphological features, molecular identification methods are a key approach to differentiate *D. betae* from neighboring species. The both neighboring species (*D. compacta and D*. *sambucusii*) of the newly discovered *Diaporthe* species were all found in China, and *D*. *sambucusii* is also discovered from Heilongjiang Province (Gao et al., 2016; Yang et al., 2018). This study indicates that new *Diaporthe* species is a result of adapting to different hosts and environmental changes during its evolutionary process.

The genus *Diaporthe*, along with its anamorph *Phomopsis*, is a highly complex group consisting of over one thousand fungal names (Index Fungroum). They are found worldwide in various ecosystems, where they can act as plant pathogens, non-pathogenic endophytes, or saprobes in terrestrial host plants (Xu et al., 2021). *Diaporthe* as pathogens have a broad host range and cause a range of diseases, such as cankers, leaf spots, dieback, rot, wilt, and blights, even though others may be host-specific (Gomes et al., 2013; Zapata et al., 2020). According to Koch’s postulates, *D. betae* is the cause of root rot in sugar beet. Through field isolations, it has been determined that *D. betae* is the causal agent of root rot in sugar beet. Additionally, *D. betae* has been found on the taproots of sugar beet plants. Considering the characteristics of this disease, we proposed *Diaporthe* root rot as a name for this disease. To our knowledge, *D. betae* is the first identified *Diaporthe* species associated with sugar beet rot in China. Notably, culturable fungi such as *F. xysporum*, *F. solani*, or *Ca. montana* can also be isolated from the same root rot samples from which the genus *Diaporthe* was isolated. *F. xysporum*, *F. solani*, and *Ca. montana* were all pathogens of sugar beet root rot that have been reported (Farhaoui et al., 2023). Previous studies have shown that sugar beet root rot pathogens, such as *P. aphanidermatum*, *R. solani*, *R*. *bataticola*, and *S*. *rolfsii*, or *F. xysporum*, *A*. *cochlioides*, and *R. solani*, in combination increased severity of disease complex of sugar beet (Majumdar et al., 2022). The role of the genus *Diaporthe* coexisting with other pathogens in invading the host requires in-depth research. Through artificial inoculation, this newly discovered pathogen could infect *Spinacia oleracea* (Supplementary Figure S4). However, the range of hosts of this pathogen in the field remains to be explored. The discovery of this new pathogenic fungus will help researchers elucidate the pathogenesis of sugar beet root rot and provide a theoretical basis for performing targeted monitoring, preventing disease and implementing control measures.

## Data Availability

The datasets presented in this study can be found in online repositories. The names of the repository/repositories and accession number(s) can be found in the article/Supplementary material.

## Footnotes

^1^ https://mafft.cbrc.jp/alignment/server/
