# Supplementary material for: Diaporthe betae sp. nov., a new species associating with sugar beet root rot in Heilongjiang Province, China
Source: Front Microbiol. 2025 Feb 26;16:1453460. doi: 10.3389/fmicb.2025.1453460 (PMC11897564; doi:10.3389/fmicb.2025.1453460)
Supplement: Supplementary file 1 [file Table_1.docx]

Supplementary Material

# Supplementary Figures and Tables

## Supplementary Figures


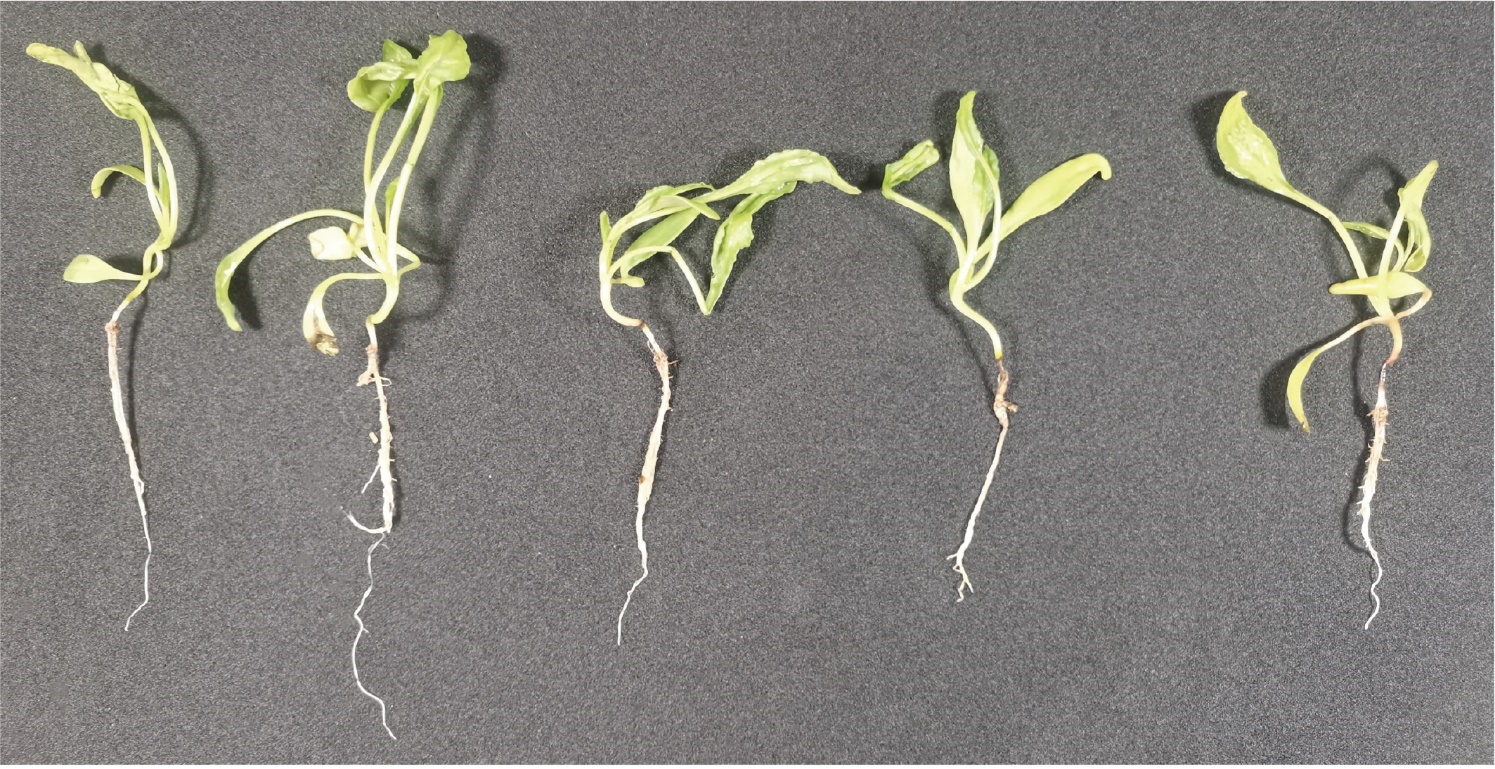


**Figure S1.** Disease severity scale for *Diaporthe betae* inoculation on sugar beet seedlings. scale 0, no disease; scale 1, one-fourth of the root was necrotic; scale 2, one-half of the root was necrotic; scale 3, three-fourths of the root was necrotic; and scale 4, the whole root was necrotic, or the seedling were completely dead


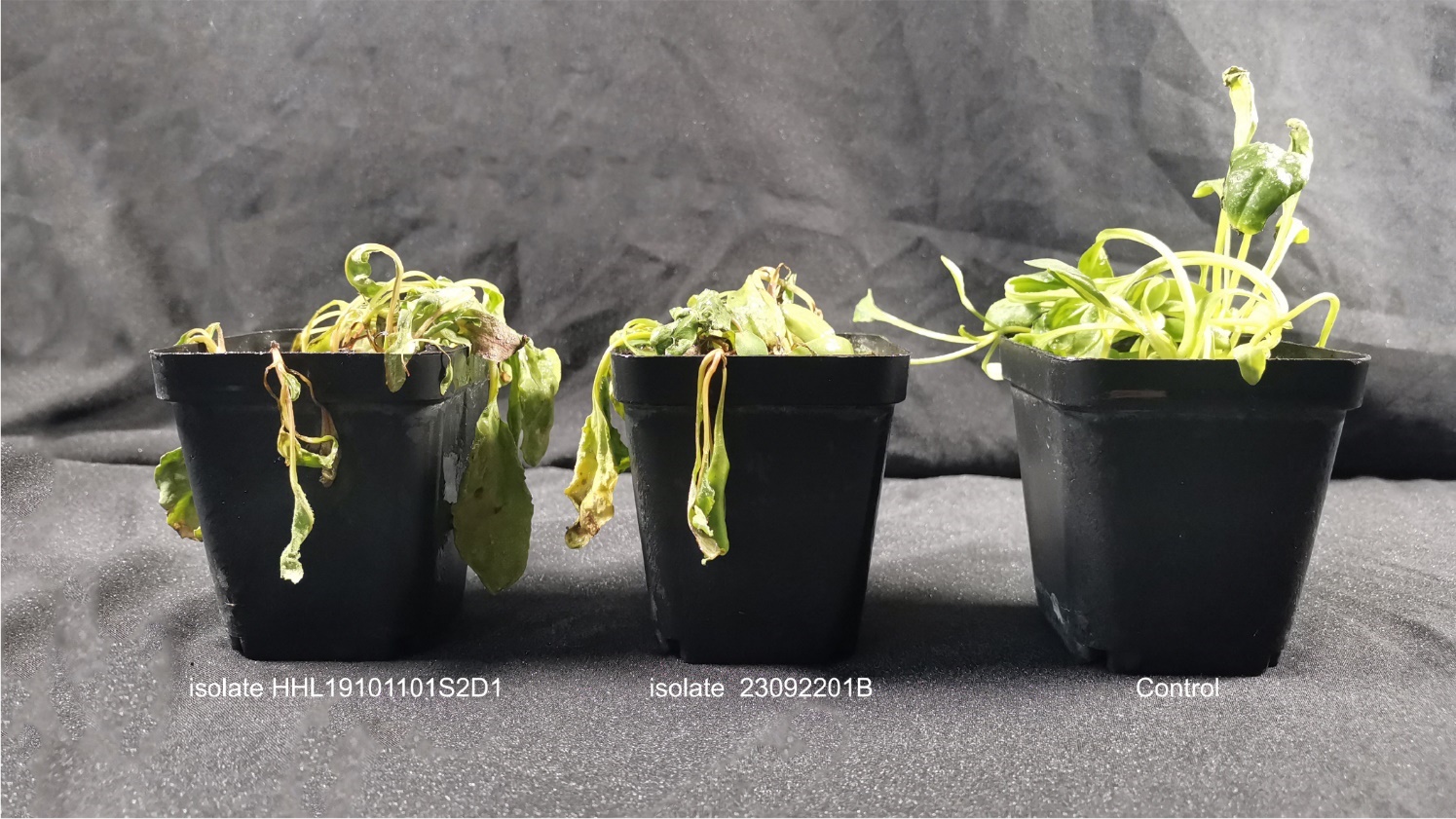


**Figure S2.** Pathogenicity of the *Diaporthe betae* pathogens


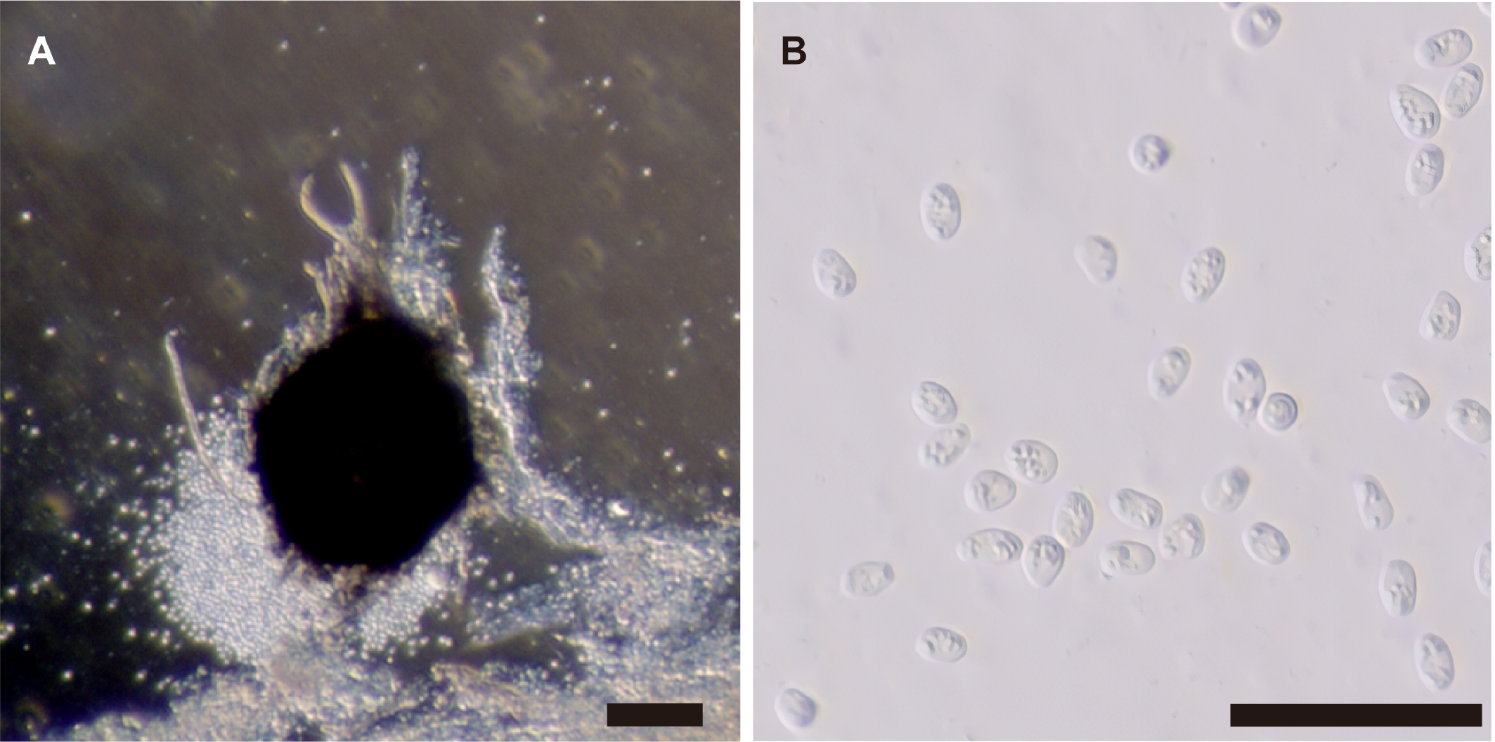


**Figure S3.** Morphology of *Phoma betae*. **(A)** Pycnidia formed on the root surface of sugar beet; **(B)** Conidia of *Phoma betae*. - Scale bars: = 20 μm


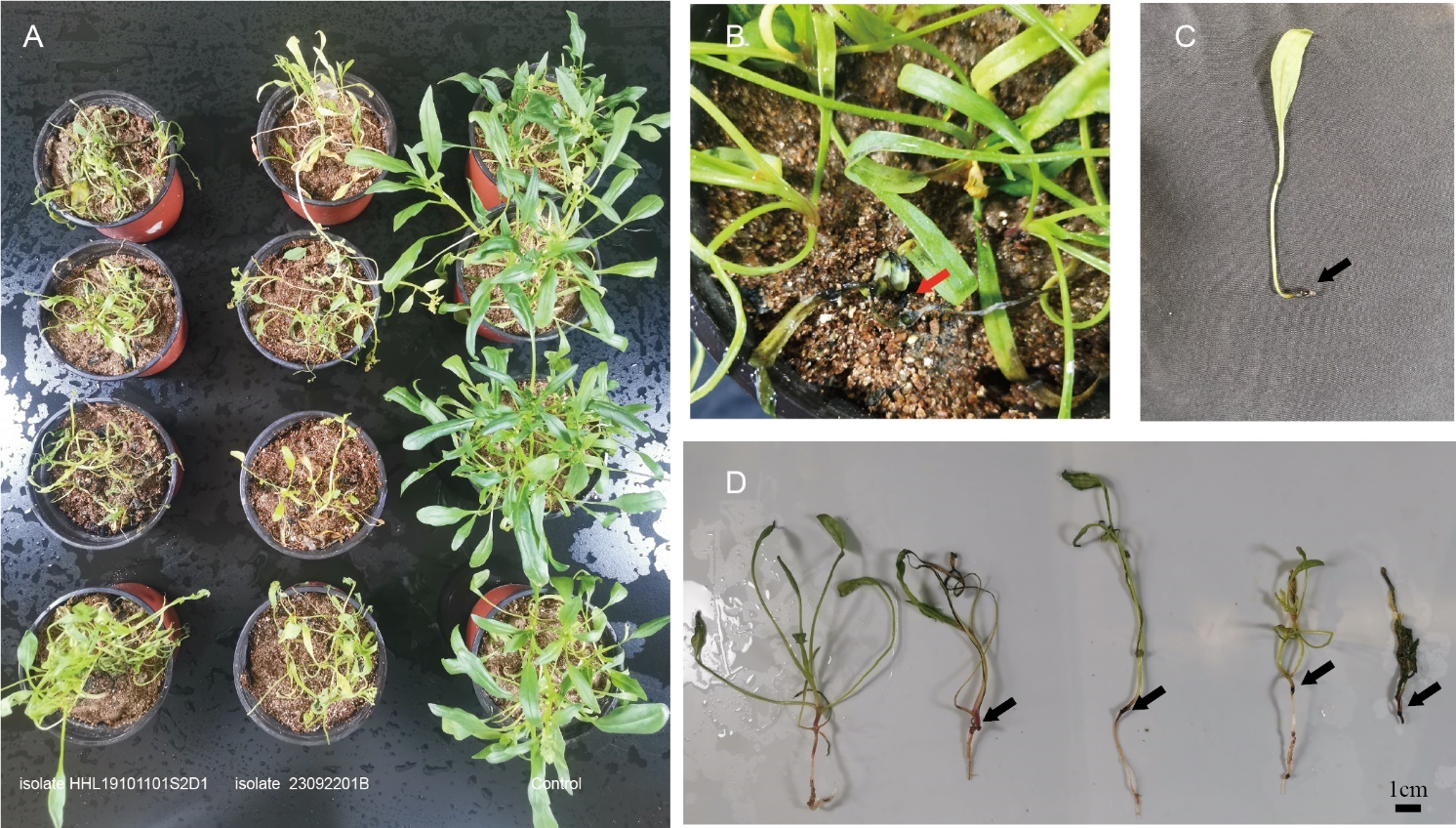


**Figure S4.** *Spinacia oleracea* infected by *Diaporthe betae*. **(B - D)** The infected part indicated by the arrow. Scal bar = 1 cm

## Supplementary Tables

**Table S1** Primers used in this study.

| Gene | Primers | Sequence (5′- 3′) | References |
| --- | --- | --- | --- |
| ITS | ITS1 | TCCGTAGGTGAACCTGCGG | (White et al., 1990) |
|  | ITS4 | TCCTCCGCTTATTGATATGC |  |
| *CAL* | CAL228F | GAGTTCAAGGAGGCCTTCTCCC | (Carbone and Kohn, 1999) |
|  | CAL737R | CATCTTTCTGGCCATCATGG |  |
| *HIS* | CYLH3F | AGGTCCACTGGTGGCAAG | (Crous et al., 2004) |
|  | H3-1b | GCGGGCGAGCTGGATGTCCTT | (Glass and Donaldson, 1995) |
| *TEF1* | EF1-728F | CATCGAGAAGTTCGAGAAGG | (Crous et al., 2004) |
|  | CylEF-R2 | CATGTTCTTGATGAA (A/G) TCACG |  |
| *TUB2* | T1 | AACATGCGTGAGATTGTAAGT | (Glass and Donaldson, 1995) |
|  | Bt2b | ACCCTCAGTGTAGTGACCCTTGGC |  |

References

White, T., Bruns, T., Lee, S., Taylor, J., Innis, M., Gelfand, D., et al. (1990). Amplification and direct sequencing of fungal ribosomal RNA genes for phylogenetics. *Pcr Protoc*. : *Guide Methods Appl*. 31**,** 315-322. doi: 10.1016/B978-0-12-372180-8.50042-1.

Carbone, I., and Kohn, L.M. (1999). A method for designing primer sets for speciation studies in filamentous ascomycetes. *Mycologia* 91(3)**,** 553-556. doi: 10.2307/3761358.

Crous, P.W., Groenewald, J.Z., Risède, J.M., Simoneau, P., and Hyde, K.D. (2006). Calonectria species and their Cylindrocladium anamorphs: species with clavate vesicles. *Stud Mycol* 55**,** 213-226. doi: 10.3114/sim.55.1.213.

Glass, N.L., and Donaldson, G.C. (1995). Development of primer sets designed for use with the PCR to amplify conserved genes from filamentous ascomycetes. *Appl Environ Microbiol* 61(4)**,** 1323-1330. doi: 10.1128/aem.61.4.1323-1330.1995.

**Table S2** Species, strain numbers, hosts, origin countries and Genbank accession numbers of fungi used in the phylogenetic study.

|  |  |  |  | GenBank accession numbers^b^ | | | | |
| --- | --- | --- | --- | --- | --- | --- | --- | --- |
| Species | Strain number | Host | Country | ITS | *CAL* | *HIS3* | *TEF1* | *TUB2* |
| *D. acaciarum* | CBS138862 * | *Vachelliatortilis* | Tanzania | KP004460 | –^a^ | KP004504 | – | KP004509 |
| *D. acericola* | MFLUCC 17-0956 * | *Acernegundo* | Italy | KY964224 | KY964137 | – | KY964180 | KY964074 |
| *D. acerigena* | CFCC 52554 * | *Acertataricum* | China | MH121489 | MH121413 | MH121449 | MH121531 | – |
| *D. acuta* | PSCG 047 * | *Pyruspyrifolia* | China | MK626957 | MK691125 | MK726161 | MK654802 | MK691225 |
|  | PSCG 046 | *Pyruspyrifolia* | China | MK626958 | MK691125 | MK726162 | MK654803 | MK691224 |
| *D. alangii* | CFCC 52556 * | *Alangiumkurzii* | China | MH121491 | MH121415 | MH121451 | MH121533 | MH121573 |
|  | CFCC 52557 | *Alangiumkurzii* | China | MH121492 | MH121416 | MH121452 | MH121534 | MH121574 |
| *D. albosinensis* | CFCC 53066 * | *Betula albosinensis* | China | MK432659 | MK442979 | MK443004 | MK578133 | MK578059 |
| *D. alleghaniensis* | CBS 495.72 * | *Betula alleghaniensis* | Canada | KC343007 | KC343249 | KC343491 | KC343733 | KC343975 |
| *D. alnea* | CBS 146.46 * | Alnus sp. | Netherlands | KC343008 | KC343250 | KC34349 | KC343734 | KC343976 |
| *D. ampelina* | CBS 114016 * | *Vitis vinifera* | France | AF230751 | JX197443 | – | AY745056 | JX275452 |
| *D. amygdali* | CBS 126679 * | *Prunus dulcis* | Portugal | KC343022 | KC343264 | KC343506 | KC343748 | KC343990 |
| *D. anacardii* | CBS 720.97 * | *Anacardium ocidentale* | East Africa | KC343024 | KC343266 | KC343508 | KC343750 | KC343992 |
|  | CBS 144610 | Unidentified leaf litter | South Africa | MK442578 | MK442651 | – | MK442692 | – |
| *D. apiculata* | CGMCC 3.17533 * | *Camellia sinensis* | China | KP267896 | – | – | KP267970 | KP293476 |
|  | LC3364 | *Camellia sinensis* | China | KP267887 | – | – | KP267961 | KP293467 |
| *D. arecae* | CBS 161.64 * | *Areca catechu* | India | KC343032 | KC343274 | KC343516 | KC343758 | KC344000 |
| *D. arengae* | CBS 114979 * | *Arenga engleri* | China | KC343034 | KC343276 | KC343518 | KC343760 | KC344002 |
| *D. aseana* | MFLUCC 12-0299a * | Unknown dead leaf | Thailand | KT459414 | KT459464 | – | KT459448 | KT459432 |
| *D. beilharziae* | BRIP 54792 * | *Indigofera australis* | Australia | JX862529 | – | – | JX862535 | KF170921 |
| *D. betulae* | CFCC 50469 * | *Betula platyphylla* | China | KT732950 | KT732997 | – | KT733016 | KT733020 |
| ***D.* *betae***^c^ | **HMPHU 3001 = HHL19101101S2D1 *** | ***Beta vulgaris* L.** | **China** | **MW882216** | **MW882219** | **MW882225** | **MW882222** | **MW882228** |
|  | **HUMCC 3268 = HHL19101101S2D2** | ***Beta vulgaris* L.** | **China** | **MW882217** | **MW882220** | **MW882226** | **MW882223** | **MW882229** |
|  | **HUMCC 3269 = HHL19101101S2D3** | ***Beta vulgaris* L.** | **China** | **MW882218** | **MW882221** | **MW882227** | **MW882224** | **MW882230** |
|  | **HUMCC 3126 = 23092201B** | ***Beta vulgaris* L.** | **China** | **PP064117** | **PP178566** | **PP178565** | **PP178567** | **PP178568** |
| *D. betulicola* | CFCC 51128 * | *Betula albosinensis* | China | KX024653 | KX024659 | KX024661 | KX024655 | KX024657 |
| *D. betulina* | CFCC 52560 * | *Betula albosinensis* | China | MH121495 | MH121419 | MH121455 | MH121537 | MH121577 |
| *D. bicincta* | CBS 121004 * | Juglans sp. | USA | KC343134 | KC343376 | KC343618 | KC343860 | KC344102 |
| *D. biconispora* | CGMCC 3.17252 * | *Citrus grandis* | China | KJ490597 | MT898460 | KJ490539 | KJ490476 | KJ490418 |
|  | YJ1 | *Sapindus mukorossi* | China | MN901242 | MT459786 | MT113117 | MT113118 | MT113116 |
| *D. biguttulata* | CGMCC 3.17248 * | *Citrus limon* | China | KJ490582 | – | KJ490524 | KJ490461 | KJ490403 |
|  | ZJUD48 | *Citrus limon* | China | KJ490583 | – | KJ490525 | KJ490462 | KJ490404 |
| *D. biguttusis* | CGMCC 3.17081 * | *Lithocarpus glabra* | China | KF576282 | – | – | KF576257 | KF576306 |
| *D. bohemiae* | CBS 143347 * | *Vitis vinifera* | Czech Republic | MG281015 | MG281710 | MG281361 | MG281536 | MG281188 |
|  | CBS 143348 | *Vitis vinifera* | Czech Republic | MG281016 | MG281711 | MG281362 | MG281537 | MG281189 |
| *D. camptothecicola* | CFCC 51632 | *Camptothecaacuminata* | China | KY203726 | KY228877 | KY228881 | KY228887 | KY228893 |
| *D. caryae* | CFCC 52563 * | *Carya illinoensis* | China | MH121498 | MH121422 | MH121458 | MH121540 | MH121580 |
|  | PSCG 520 | *Pyruspyrifolia* | China | MK626952 | MK691200 | MK726202 | MK654895 | MK691315 |
| *D. castaneae-mollissimae* | DNP 128 * | *Castanea mollissima* | China | JF957786 | JX197430 | – | JX275401 | JX275438 |
| *D. celastrina* | CBS 139.27 * | Celastrus sp. | USA | KC343047 | KC343289 | KC343531 | KC343773 | KC344015 |
| *D. celeris* | CBS 143349 * | *Vitis vinifera* | Czech Republic | MG281017 | MG281712 | MG281363 | MG281538 | MG281190 |
| *D. cercidis* | CFCC 52565 * | *Cercis chinensis* | China | MH121500 | MH121424 | MH121460 | MH121542 | MH121582 |
|  | CFCC 52566 | *Cercis chinensis* | China | MH121501 | MH121425 | MH121461 | MH121543 | MH121583 |
| *D. chensiensis* | CFCC 52567 * | *Abies chensiensis* | China | MH121502 | MH121426 | MH121462 | MH121544 | MH121584 |
| *D. cinnamomi* | CFCC 52569 * | Cinnamomum sp. | China | MH121504 | – | MH121464 | MH121546 | MH121586 |
| *D. citri* | CBS 135422 * | Citrus sp. | USA | KC843311 | KC843157 | – | KC843071 | KC843187 |
|  | ZJUD1 | *Citrus reticulata* | China | JQ954654 | – | KJ490514 | JQ954671 | KJ490395 |
| *D. citriasiana* | CBS134240 * | *Citrus unshiu* | China | JQ954645 | KC357491 | KJ490515 | JQ954663 | KC357459 |
|  | ZJUD82 | *Citrus maxima* | China | KJ490617 | – | KJ490559 | KJ490496 | KJ490438 |
| *D. citrichinensis* | CBS 134242 * | *Citrus unshiu* | China | JQ954648 | KC357494 | KJ490516 | JQ954666 | KJ490396 |
| *D. collariana* | MFLUCC 17-2636 * | *Magnolia champaca* | Thailand | MG806115 | MG783042 | – | MG783040 | MG783041 |
| *D. compacta* | CGMCC 3.17536 * | *Camellia sinensis* | China | KP267854 | – | KP293508 | KP267928 | KP293434 |
|  | LC3084 | *Camellia sinensis* | China | KP267855 | – | KP293509 | KP267929 | KP293435 |
|  | LC3078 | *Camellia sinensis* | China | KP267850 | – | KP293504 | KY011839 | KP293430 |
|  | ZJUE0380 | *Citrus inensis* | China | OP218129 | OP265456 | OP265520 | OP265584 | OP265648 |
|  | ZJUE0403 | *Citrus reticulata* | China | OP218130 | OP265457 | OP265521 | OP265585 | OP265649 |
| *D. conica* | CFCC 52571 * | *Alangium chinense* | China | MH121506 | MH121428 | MH121466 | MH121548 | MH121588 |
| *D. convolvuli* | CBS 124654 * | *Convolvulus arvensis* | Turkey | KC343054 | KC343296 | KC343538 | KC343780 | KC344022 |
| *D. coryli* | CFCC 53083 * | *Corylus mandshurica* | China | MK432661 | MK442981 | MK443006 | MK578135 | MK578061 |
|  | CFCC 53084 | *Corylus mandshurica* | China | MK432662 | MK442982 | MK443007 | MK578136 | MK578062 |
| *D. cotoneastri* | CBS 439.82 * | *Cotoneastersp.* | UK | FJ889450 | JX197429 | – | GQ250341 | JX275437 |
| *D. diospyricola* | CBS 136552 * | *Diospyros whyteana* | South Africa | KF777156 | – | – | – | – |
| *D. discoidispora* | CGMCC 3.17255 * | *Citrus unshiu* | China | KJ490624 | – | KJ490566 | KJ490503 | KJ490445 |
| *D. ellipicola* | CGMCC 3.17084 * | *Lithocarpus glabra* | China | KF576270 | – | – | KF576245 | KF576294 |
| *D. endophytica* | CBS 133811 * | *Schinus terebinthifolius* | Brazil | KC343065 | KC343307 | KC343549 | KC343791 | KC344033 |
| *D. eres* | AR5193 * | *Ulmus* sp. | Germany | KJ210529 | KJ434999 | KJ420850 | KJ210550 | KJ420799 |
| *D. eugeniae* | CBS 444.82 * | *Eugenia aromatica* | Indonesia | KC343098 | KC343340 | KC343582 | KC343824 | KC344066 |
| *D. fraxini-angustifoliae* | BRIP 54781 * | *Fraxinus angustifolia* | Australia | JX862528 | – | – | JX862534 | KF170920 |
| *D. fraxinicola* | CFCC 52582 * | *Fraxinus chinensis* | China | MH121517 | MH121435 | – | MH121559 | – |
| *D. fukushii* | MAFF 625034 | *Pyrus pyrifolia* | Japan | JQ807469 | – | – | JQ807418 | – |
| *D. fulvicolor* | PSCG 051 * | *Pyrus pyrifolia* | China | MK626859 | MK691132 | MK726163 | MK654806 | MK691236 |
|  | PSCG 057 | *Pyrus pyrifolia* | China | MK626858 | MK691131 | MK726164 | MK654810 | MK691233 |
| *D. fusicola* | CGMCC 3.17087 * | *Lithocarpus glabra* | China | KF576281 | KF576233 | – | KF576256 | KF576305 |
|  | PSCG 015 | *Pyruspyrifolia* | China | MK626915 | MK691210 | MK726254 | MK654861 | MK691320 |
| *D. ganjae* | CBS 180.91 * | *Cannabis sativa* | USA | KC343112 | KC343354 | KC343596 | KC343838 | KC344080 |
|  | PSCG489 |  |  | MK626955 | MK691202 | MK726204 | MK654897 | MK691287 |
| *D. guangxiensis* | JZB320094 * | *Vitis vinifera* | China | MK335772 | MK736727 | – | MK523566 | MK500168 |
|  | JZB320093 | *Vitis vinifera* | China | MK335771 | MK736726 | – | MK523565 | MK500167 |
| *D. gulyae* | BRIP 54025 * | *Helianthus annuus* | Australia | JF431299 | – | – | JN645803 | – |
| *D. helicis* | CBS 138596 * | *Hedera helix* | France | KJ210538 | KJ435043 | KJ420875 | KJ210559 | KJ420828 |
| *D. heliconiae* | SAUCC194.77 * | *Heliconia metallica* | China | MT822605 | MT855684 | MT855573 | MT855917 | MT855802 |
|  | SAUCC194.75 | *Heliconia metallica* | China | MT822603 | MT855682 | MT855571 | MT855915 | MT855800 |
| *D. heterophyllae* | CBS 143769 * | *Acacia heterohpylla* | France | MG600222 | MG600218 | MG600220 | MG600224 | MG600226 |
| *D. hispaniae* | CBS 143351 * | *Vitis vinifera* | Spain | MG281123 | MG281820 | MG281471 | MG281644 | MG281296 |
| *D. hongkongensis* | CBS 115448 * | *Dichroa febrífuga* | China | KC343119 | KC343361 | KC343603 | KC343845 | KC344087 |
| *D. hubeiensis* | JZB320123 * | *Vitis vinifera* | China | MK335809 | MK500235 | – | MK523570 | MK500148 |
|  | JZB320122 | *Vitis vinifera* | China | MK335808 | MK500234 | – | MK523569 | MK500147 |
| *D. incompleta* | CGMCC 3.18288 * | *Camellia sinensis* | China | KX986794 | KX999289 | KX999265 | KX999186 | KX999226 |
| *D. inconspicua* | CBS 133813 * | *Maytenusilicifolia* | Brazil | KC343123 | KC343365 | KC343607 | KC343849 | KC344091 |
| *D. infecunda* | CBS 133812 * | *Schinus terebinthifolius* | Brazil | KC343126 | KC343368 | KC343610 | KC343852 | KC344094 |
| *D. isoberliniae* | CPC 22549 * | *Isoberlinia angolensis* | Zambia | KJ869133 | – | – | – | KJ869245 |
| *D. juglandicola* | CFCC 51134 * | *Juglans mandshurica* | China | KU985101 | KX024616 | KX024622 | KX024628 | KX024634 |
| *D. kadsurae* | CFCC 52586 * | *Kadsura longipedunculata* | China | MH121521 | MH121439 | MH121479 | MH121563 | MH121600 |
| *D. kochmanii* | BRIP 54033 * | *Helianthus annuus* | Australia | JF431295 | – | – | JN645809 | – |
| *D. kongii* | BRIP 54031 * | *Helianthus annuus* | Australia | JF431301 | – | – | JN645797 | – |
| *D. limonicola* | CBS 142549 * | *Citrus limon* | Malta | MF418422 | MF418256 | MF418342 | MF418501 | MF418582 |
|  | CBS 142550 | *Citrus limon* | Malta | MF418423 | MF418257 | MF418343 | MF418502 | MF418583 |
| *D. litchicola* | BRIP 54900 * | *Litchi chinensis* | Australia | JX862533 | – | – | JX862539 | KF170925 |
| *D. litchii* | SAUCC194.22 * | *Litchi chinensis* | China | MT822550 | MT855635 | MT8555 | MT855863 | MT855747 |
| *D. lithocarpus* | CGMCC 3.15175 * | *Lithocarpus glabra* | China | KC153104 | KF576235 | – | KC153095 | KF576311 |
|  | CGMCC 3.17098 | *Lithocarpus glabra* | China | KF576276 | KF576228 | – | KF576251 | KF576300 |
| *D. leucospermi* | CAA971 | *Vaccinium corymbosum* | Location | – | MT051860 | MT051896 | MT051933 | MT051968 |
| *D. longicicola* | CGMCC 3.17089 * | *Lithocarpus glabra* | China | KF576267 | – | – | KF576242 | KF576291 |
| *D. longicolla* | FAU599 * | *Glycine max* | USA | KJ590728 | KJ612124 | KJ659188 | KJ590767 | KJ610883 |
| *D. lonicerae* | MFLUCC 17-0963 * | *Lonicera* sp. | Italy | KY964190 | KY964116 | – | KY964146 | KY964073 |
| *D. machili* | SAUCC 194.111 * | *Machilus pingii* | China | MT822639 | MT855718 | MT855606 | MT855951 | MT855836 |
|  | SAUCC194.69 | *Pometia pinnata* | China | MT822597 | MT855677 | MT855565 | MT855909 | MT855794 |
| *D. mahothocarpus* | CGMCC 3.15181 * | *Lithocarpus glabra* |  | KC153096 | KT459461 | – | KC153087 | KF576312 |
| *Diaporthe manihotia* | CBS 505.76 * | *Manihot utilissima* | Rwanda | KC343138 | KC343380 | KC343622 | KC343864 | KC344106 |
|  | MFLU:19-2826 | *Camellia* sp. | China | MT012296 | MW014358 | – | MW014359 | MW01892 |
| *D. maritima* | NB365-71 * | *Picea rubens* | Canada | KU552025 | MN136126 | MN136175 | KU552023 | KU574615 |
| *D. masirevicii* | BRIP 57892a * | *Helianthus annuus* | Australia | KJ197276 | – | – | KJ197239 | KJ197257 |
| *D. melitensis* | CBS 142551 * | *Citrus limon* | Malta | MF418424 | MF418258 | MF418344 | MF418503 | MF418584 |
|  | CPC 27875 | *Citrus limon* | Malta | MF418425 | MF418259 | MF418345 | MF418504 | MF418585 |
| *D. middletonii* | BRIP 54884e * | *Rapistrum rugostrum* | Australia | KJ197286 | – | – | KJ197248 | KJ197266 |
| *D. miriciae* | BRIP 54736j * | *Helianthus annuus* | Australia | KJ197282 | – | – | KJ197244 | KJ197262 |
| *D. momicola* | MFLUCC 16-0113 * | *Prunus persica* | China | KU557563 | KU557611 | – | KU557631 | KU557587 |
| *D. multigutullata* | CGMCC 3.17258 * | *Citrus maxima* | China | KJ490633 | – | KJ490575 | KJ490512 | KJ490454 |
| *D. musigena* | CBS 129519 * | *Musa* sp. | Australia | KC343143 | KC343385 | KC343627 | KC343869. | KC344111 |
| *D. neilliae* | CBS 144. 27 * | *Spiraea* sp. | USA | KC343144 | KC343386 | KC343628 | KC343870 | KC344112 |
| *D. nobilis* | CBS 200.39 | *Laurus nobilis* | Germany | KC343151 | KC343393 | KC343635 | KC343877 | KC344119 |
| *D. novem* | CBS 127270 * | *Glycine max* | Croatia | KC343156 | KC343398 | KC343640 | KC343882 | KC344124 |
|  | CBS 127271 | *Glycine max* | Croatia | KC343157 | KC343399 | KC343641 | KC343883 | KC344125 |
| *D. oncostoma* | CBS 589.78 | *Robinia pseudoacacia* | France | KC343162 | KC343404 | KC343646 | KC343888 | KC344130 |
| *D. oraccinii* | CGMCC 3.17531 * | *Camellia sinensis* | China | KP267863 | – | KP293517 | KP267937 | KP293443 |
| *D. ovalispora* | CGMCC 3.17256 * | *Citrus limon* | China | KJ490628 | – | KJ490570 | KJ490507 | KJ490449 |
| *D. oxe* | CBS 133186 | *Maytenus ilicifolia* | Brazil | KC343164 | KC343406 | KC343648 | KC343890 | KC344132 |
| *D. oxe* | LGMF939 | *Maytenus ilicifolia* | Location | KC343167 | KC343409 | KC343651 | KC343893 | KC344135 |
| *D. padina* | CFCC 52590 * | *Padus racemosa* | China | MH121525 | MH121443 | MH121483 | MH121567 | MH121604 |
| *D. pascoei* | BRIP 54847 * | *Persea americana* | Australia | JX862532 | – | – | JX862538 | KF170924 |
| *D. passifloricola* | CBS 141329 * | *Passiflora foetida* | Malaysia | KX228292 | – | KX228367 | – | KX228387 |
| *D. penetriteum* | CGMCC 3.17532 * | *Camellia sinensis* | China | KP714505 | – | KP714493 | KP714517 | KP714529 |
| *D. perseae* | CBS 151.73 * | *Persea gratissima* | Netherlands | KC343173 | KC343415 | KC343657 | KC343899 | KC344141 |
| *D. pescicola* | MFLUCC 16-0105 * | *Prunus persica* | China | KU557555 | KU557603 | – | KU557623 | KU557579 |
|  | MFLUCC 16-0106 | *Prunus persica* | China | KU557556 | KU557604 | – | KU557624 | KU557580 |
| *D. phragmitis* | CBS 138897 * | *Phragmites australis* | China | KP004445 | – | KP004503 | – | KP004507 |
| *D. phaseolorum* | CBS 116019 | *Caperonia palustris* | USA | KC343175 | KC343417 | KC343659 | KC343901 | KC344143 |
|  | CBS 116020 | *Aster exilis* | USA | KC343176 | KC343418 | KC343660 | KC343902 | KC344144 |
| *D. podocarpi-macrophylli* | CGMCC 3.18281 * | *Podocarpus macrophyllus* | China | KX986774 | KX999278 | KX999246 | KX999167 | KX999207 |
| *D. pometiae* | SAUCC 194.72 * | *Pometia pinnata* | China | MT822600 | MT855679 | MT855568 | MT855912 | MT855797 |
|  | SAUCC 194.19 | *Persea americana* | China | MT822547 | MT855632 | MT855516 | MT855861 | MT855744 |
| *D. pseudomangiferae* | CBS 101339 * | *Mangifera indica* | Dominican Republic | KC343181 | KC343423 | KC343665 | KC343907 | KC344149 |
| *D. pseudophoenicicola* | CBS 462.69 * | *Phoenix dactylifera* | Spain | KC343184 | KC343426 | KC343668 | KC343910 | KC344152 |
| *D. pterocarpi* | MFLUCC 10-0571 * | *Pterocarpus indicus* | Thailand | JQ619899 | JX197451 | – | JX275416 | JX275460 |
| *D. pterocarpicola* | MFLUCC 10-0580a * | *Pterocarpus indicus* | Thailand | JQ619887 | JX197433 | – | JX275403 | JX275441 |
| *D. pulla* | CBS 338.89 * | *Hedera helix* | Yugoslavia | KC343152 | KC343394 | KC343636 | KC343878 | KC344120 |
| *D. pyracanthae* | CAA 483 * | *Pyracantha coccinea* | Portugal | KY435635 | KY435656 | KY435645 | KY435625 | KY435666 |
| *D. rosae* | MFLUCC 17-2658 * | *Rosa sp.* | Thailand | MG828894 | – | – | – | MG843878 |
| *D. rosicola* | MFLU 17-0646 * | *Rosa* sp. | UK | MG828895 |  |  | MG829270 | MG843877 |
| *D. rostrata* | CFCC 50062 * | *Juglans mandshurica* | China | KP208847 | KP208849 | KP208851 | KP208853 | KP208855 |
| *D. saccarata* | CBS 116311 * | *Protea repens* | South Africa | KC343190 | KC343432 | KC343674 | KC343916 | KC344158 |
| *D. sackstonii* | BRIP 54669b * | *Helianthus annuus* | Australia | KJ197287 | – | – | KJ197249 | KJ197267 |
| *D. sambucusii* | CFCC 51986 * | *Sambucus williamsii* | China | KY852495 | KY852499 | KY852503 | KY852507 | KY852511 |
|  | CFCC 51987 | *Sambucus williamsii* | China | KY852496 | KY852500 | KY852504 | KY852508 | KY852512 |
| *D. sclerotioides* | CBS:296.67 | *Cucumis sativus* | Netherlands | MH858974. | KC343435 | KC343677 | KC343919 | KC344161 |
| *D. sclerotioides* | CBS 710.76 | *Cucumis sativus* | Netherlands | KC343194 | KC343436 | KC343678 | KC343920 | KC344162 |
| *D. schisandrae* | CFCC 51988 * | *Schisandra chinensis* | China | KY852497 | KY852501 | KY852505 | KY852509 | KY852513 |
| *D. schoeni* | MFLU 15-1279 * | *Schoenus nigricans* | Italy | KY964226 | KY964139 | – | KY964182 | KY964109 |
| *D. sennae* | CFCC 51636 * | *Senna bicapsularis* | China | KY203724 | KY228875 | KY228879 | KY228885 | KY228891 |
| *D. sennicola* | CFCC 51634 * | *Senna bicapsularis* | China | KY203722 | KY228873 | – | KY228883 | KY228889 |
|  | CFCC 51635 | *Senna bicapsularis* | China | KY203723 | KY228874 | KY228880 | KY228884 | KY228890 |
| *D. serafiniae* | BRIP 55665a * | *Helianthus annuus* | Australia | KJ197274 | – | – | KJ197236 | KJ197254 |
| *D. shaanxiensis* | CFCC 53106 * | *On branches of liana* | China | MK432654 | MK442976 | MK443001 | MK578130 | – |
|  | CFCC 53107 | *On branches of liana* | China | MK432655 | MK442977 | MK443002 | MK57813 | – |
| *D. siamensis* | MFLUCC 10-573a * | *Dasymaschalon sp.* | Thailand | JQ619879 | – | – | JX275393 | JX275429 |
| *D. sojae* | FAU 635 * | *Glycine max* | USA | KJ590719 | KJ612116 | KJ659208 | KJ590762 | KJ610875 |
| *D. spinosa* | PSCG 383 * | *Pryuspyrifolia* | China | MK626849 | MK691129 | MK726156 | MK654811 | MK691234 |
| *D. sterilis* | CBS 136969 * | *Vaccinium corymbosum* | Italy | KJ160579 | KJ160548 | MF418350 | KJ160611 | KJ160528 |
|  | CBS 136970 | *Vaccinium corymbosum* | Italy | KJ160580 | KJ160549 |  | KJ160612 | KJ160529 |
| *D. subclavata* | CGMCC 3.17257 * | *Citrus unshiu* | China | KJ490630 | – | KJ490572 | KJ490509 | KJ490451 |
| *D. subellipicola* | KUMCC 17-0153 * | *On deadwood* | China | MG746632 | – |  | MG746633 | MG746634 |
| *D. taoicola* | MFLUCC 16-0117 * | *Prunus persica* | China | KU557567 | – |  | KU557635 | KU557591 |
|  | MFLUCC 16-0118 | *Prunus persica* | China | KU557568 | – | – | KU557636 | KU557591 |
| *D. tectonae* | MFLUCC 12-0777 * | *Tectona grandis* | China | KU712430 | KU749345 |  | KU749359 | KU743977 |
| *D. tectonendophytica* | MFLUCC 13-0471 * | *Tectona grandis* | Thailand | KU712439 | KU749354 | KX999266 | KU749367 | KU743986 |
| *D. tectonigena* | MFLUCC 12-0767 * | *Tectona grandis* | Thailand | KU712429 | KU749358 |  | KU749371 | KU743976 |
| *D. thunbergiicola* | MFLUCC 12-0033 * | *Thunbergia laurifolia* | Thailand | KP715097 | – | – | KP715098 | – |
| *D. tibetensis* | CFCC 51999 * | *Juglandis regia* | China | MF279843 | MF279888 | MF279828 | MF279858 | MF279873 |
| *D. tulliensis* | BRIP 62248a * | *Theobroma cacao fruit* | Australia | KR936130 | – |  | KR936133 | KR936132 |
| *D. ueckerae* | FAU 656 * | *Cucumis melo* | USA | KJ590726 | KJ612122 | KJ659215 | KJ590747 | KJ610881 |
| *D. ukurunduensis* | CFCC 52592 * | *Acer ukurunduense* | China | MH121527 | MH121445 | MH121485 | MH121569 | – |
| *D. undulata* | CGMCC 3.18293 * | *Leaf of unknown host* | China-Laos border | KX986798 | – | KX999269 | KX999190 | KX999230 |
| *D. unshiuensis* | CGMCC 3.17569 * | *Citrus unshiu* | China | KJ490587 | – | KJ490529 | KJ490466 | KJ490408 |
|  | CGMCC 3.17568 | *Citrus japonica* | China | KJ490586 | – | KJ490528 | KJ490465 | KJ490407 |
| *D. vaccinii* | CBS 160.32 * | *Oxycoccus macrocarpos* | USA | KC343228 | KC343470 | KC343712 | KC343954 | KC344196 |
| *D. velutina* | CGMCC 3.18286 * | *Neolitsea* sp. | China | KX986790 | – |  | KX999182 | KX999223 |
|  | PSCG 134 | *Pyruspyrifolia* | China | MK626918 | MK691173 | MK726205 | MK654853 | MK691243 |
| *D. virgiliae* | CMW40748 | *Virgilia oroboides* | South Africa | KP247566 | – | – | – | KP247575 |
| *D. xishuangbanica* | CGMCC 3.18282 * | *Camellia sinensis* | China | KX986783 | – | KX999255 | KX999175 | KX999216 |
| *D. yunnanensis* | CGMCC 3.18289 * | *Coffea* sp. | China | KX986796 | KX999290 | KX999267 | KX999188 | KX999228 |
| *D. zaofenghuang* | CGMCC 3.20271 * | *Prunus persica* | China | MW477883 | MW480867 | MW480863 | MW480871 | MW480875 |
|  | TZFH3 | *Prunus persica* | China | MW477884 | MW480868 | MW480864 | MW480872 | MW480876 |
| *Diaporthella corylina* | CBS 121124 * | *Corylus* sp. | China | KC343004 | KC343246 | KC343488 | KC343730 | KC343972 |

^a^ – represent not applicable.

^b^ ITS, internal transcribed spacer; *CAL*, calmodulin; *HIS3*, histone H3; *TEF1*, translation elongation factor 1-alpha; *TUB2*, beta-tubulin.

^C^ New species are bold

* represent ex-type strains

**Table S3** Nucleotide substitution models used in the phylogenetic analyses.

| Loci/Genes | Best-fit model for ML Analysis | Best-fit model for BI Analysis |
| --- | --- | --- |
| ITS | TIM2+F+R10 | GTR+F+I+G4 |
| *CAL* | TIM+F+I+G4 | SYM+G4 |
| *HIS* | GTR+F+I+G4 | GTR+F+I+G4 |
| *TUB2* | GTR+F+R4 | GTR+F+I+G4 |
| *TEF1* | TPM3u+F+R6 | HKY+F+I+G4 |

BI, Bayesian inference; ML, maximum likelihood.

**Table S4** the similarity (%) with β-tubulin 2 sequences among 4 isolates and reference strains

|  | βeta-tubulin 2 (*tub2*) | | | |
| --- | --- | --- | --- | --- |
|  | HHL19101101S2D1  (MW882228) | HHL19101101S2D2  (MW882229) | HHL19101101S2D3  (MW882230) | 23092201B  (PP178568) |
| HHL19101101S2D1^1^  (MW882228)^2^ | 876/876(100%)^3^ | 873/876(99%) | 872/876(99%) | 537/541(99%) |
| HHL19101101S2D2  (MW882229) | 872/876(99%)^3^ | 876/876(100%) | 872/876(99%) | 537/541(99%) |
| HHL19101101S2D3  (MW882230) | 871/876(99%) | 872/876(99%) | 876/876(100%) | 539/541(99%) |
| 23092201B  (PP178568) | 538/544(99%) | 538/544(99%) | 540/544(99%) | 557/557(100%) |
| *Diaporthe compacta* strain LC3083  (KP293434) | 360/364(99%) | 360/364(99%) | 360/364(99%) | 360/364(99%) |
| *Diaporthe compacta* strain LC3084  (KP293435) | 360/364(99%) | 360/364(99%) | 360/364(99%) | 360/364(99%) |
| *Diaporthe compacta* strain LC3078  (KP293430) | 360/364(99%) | 360/364(99%) | 360/364(99%) | 360/364(99%) |
| *Diaporthe compacta* isolate ZJUE0380  (OP265648) | 519/540(96%) | 519/540(96%) | 521/540(96%) | 521/540(96%) |
| *Diaporthe compacta* isolate ZJUE0403  (OP265649) | 526/540(97%) | 526/540(97%) | 528/540(98%) | 528/540(98%) |
| *Diaporthe sambucusii* strain CFCC 51986  (KY852511) | 709/722(98%) | 709/722(98%) | 709/722(98%) | 443/455(97%) |
| *Diaporthe sambucusii* strain CFCC51987  (KY852512) | 709/722(98%) | 709/722(98%) | 709/722(98%) | N/A^4^ |
| *Diaporthe ganjae* strain CBS 180.91  (KC344080) | 712/734(97%) | 712/734(97%) | 712/734(97%) | 421/427(99%) |
| *Diaporthe manihotia* strain CBS 505.76  (KC344106) | 701/722(97%) | 701/722(97%) | 701/722(97%) | 438/455(96%) |
| *Diaporthe manihotia* culture MFLU:19-2826  (MW018927) | 688/711(97%) | 688/711(97%) | 688/711(97%) | 436/455(96%) |

^1^ 4 isolates and GenBank accession Numbers.

^2^ the reference strains and GenBank accession Numbers.

^3^ Identities.

^4^ N/A: information are not available.

**Table S5** the similarity (%) with calmodulin sequences among 4 isolates and reference strains

|  | calmodulin (cmd*A*) | | | |
| --- | --- | --- | --- | --- |
|  | HHL19101101S2D1  (MW882219) | HHL19101101S2D2  (MW882220) | HHL19101101S2D3  (MW882221) | 23092201B  (PP178566) |
| HHL19101101S2D1^1^  (MW882219)^2^ | 541/541(100%)^3^ | 538/540(99%) | 539/541(99%) | 537/541(99%) |
| HHL19101101S2D2  (MW882220) | 538/540(99%) | 538/538(100%) | 538/538(100%) | 534/540(99%) |
| HHL19101101S2D3  (MW882221) | 539/541(99%) | 538/538(100%) | 539/539(100%) | 535/541(99%) |
| 23092201B  (PP178566) | 537/541(99%) | 534/540(99%) | 535/541(99%) | 539/539(100%) |
| *Diaporthe compacta* strain LC3083  (N/A) | N/A^4^ | N/A | N/A | N/A |
| *Diaporthe compacta* strain LC3084  (N/A) | N/A | N/A | N/A | N/A |
| *Diaporthe compacta* strain LC3078  (N/A) | N/A | N/A | N/A | N/A |
| *Diaporthe compacta* isolate ZJUE0380  (OP265456) | 499/512(97%) | 499/512(97%) | 499/512(97%) | 498/511(97%) |
| *Diaporthe compacta* isolate ZJUE0403  (OP265457) | 498/512(97%) | 498/512(97%) | 498/512(97%) | 497/511(97%) |
| *Diaporthe sambucusii* strain CFCC51986  (KY852499) | 494/501(99%) | 494/501(99%) | 494/501(99%) | 494/501(99%) |
| *Diaporthe sambucusii* strain CFCC51987  (KY852500) | 494/501(99%) | 494/501(99%) | 494/501(99%) | N/A |
| *Diaporthe ganjae* strain CBS 180.91  (KC343354) | 502/513(98%) | 502/513(98%) | 502/513(98%) | 501/512(98%) |
| *Diaporthe manihotia* strain CBS 505.76  (KC343380) | 490/511(96%) | 490/511(96%) | 490/511(96%) | 490/511(96%) |
| *Diaporthe manihotia* culture MFLU:19-2826  (MW014358) | 488/511(95%) | 488/511(95%) | 488/511(95%) | 488/511(95%) |

^1^ 4 isolates and GenBank accession Numbers.

^2^ the reference strains and GenBank accession Numbers.

^3^ Identities.

^4^ N/A: information are not available.

**Table S6** the similarity (%) with histone *H3* sequences among 4 isolates and reference strains

|  | histone H3 (*his3*) | | | |
| --- | --- | --- | --- | --- |
|  | HHL19101101S2D1  (MW882225) | HHL19101101S2D2  (MW882226) | HHL19101101S2D3  (MW882227) | 23092201B  (PP178565) |
| HHL19101101S2D1^1^  (MW882225)^2^ | 502/502(100%)^3^ | 501/502(99%) | 502/502(100%) | 490/491(99%) |
| HHL19101101S2D2  (MW882226) | 501/502(99%) | 503/503(100%) | 503/504(99%) | 490/491(99%) |
| HHL19101101S2D3  (MW882227) | 502/502(100%) | 503/504(99%) | 504/504(100%) | 490/491(99%) |
| 23092201B  (PP178565) | 490/491(99%) | 490/491(99%) | 490/491(99%) | 491/491(100%) |
| *Diaporthe compacta* strain LC3083  (KP293508) | 434/440(99%) | 434/440(99%) | 434/440(99%) | 433/440(98%) |
| *Diaporthe compacta* strain LC3084  (KP293509) | 434/440(99%) | 434/440(99%) | 434/440(99%) | 433/440(98%) |
| *Diaporthe compacta* strain LC3078  (KP293504) | 434/440(99%) | 434/440(99%) | 434/440(99%) | 433/440(98%) |
| *Diaporthe compacta* isolate ZJUE0380  (OP265520) | 467/473(99%) | 467/473(99%) | 467/473(99%) | 466/473(99%) |
| *Diaporthe compacta* isolate ZJUE0403  (OP265521) | 465/473(98%) | 465/473(98%) | 465/473(98%) | 464/473(98%) |
| *Diaporthe sambucusii* strain CFCC51986  (KY852503) | 460/473(97%) | 460/473(97%) | 460/473(97%) | 459/473(97%) |
| *Diaporthe sambucusii* strain CFCC51987  (KY852504) | 460/473(97%) | 460/473(97%) | 460/473(97%) | 459/473(97%) |
| *Diaporthe ganjae* strain CBS 180.91  (KC343596) | 437/444(98%) | 437/444(98%) | 437/444(98%) | 459/474(97%) |
| *Diaporthe manihotia* strain CBS 505.76  (KC343622) | 459/476(96%) | 459/476(96%) | 459/476(96%) | 458/476(96%) |
| *Diaporthe manihotia* culture MFLU:19-2826  (N/A) | N/A^4^ | N/A | N/A | N/A |

^1^ 4 isolates and GenBank accession Numbers.

^2^ the reference strains and GenBank accession Numbers.

^3^ Identities.

^4^ N/A: information are not available.

**Table S7** the similarity (%) with translation elongation factor 1-alpha (*tef1*) sequences among 4 isolates and reference strains

|  | translation elongation factor 1-alpha (*tef1*) | | | |
| --- | --- | --- | --- | --- |
|  | HHL19101101S2D1  (MW882222) | HHL19101101S2D2  (MW882223) | HHL19101101S2D3  (MW882224) | 23092201B  (PP178567) |
| HHL19101101S2D1^1^  (MW882222)^2^ | 591/591(100%)^3^ | 587/591(99%) | 584/591(99%) | 560/566(99%) |
| HHL19101101S2D2  (MW882223) | 587/591(99%) | 591/591(100%) | 588/591(99%) | 564/566(99%) |
| HHL19101101S2D3  (MW882224) | 584/591(99%) | 588/591(99%) | 591/591(100%) | 578/585(99%) |
| 23092201B  (PP178567) | 560/566(99%) | 564/566(99%) | 578/585(99%) | 598/598(100%) |
| *Diaporthe compacta* strain LC3083  (KP267928) | 324/338(96%) | 327/338(97%) | 327/338(97%) | 327/338(97%) |
| *Diaporthe compacta* strain LC3084  (KP267929) | 324/338(96%) | 327/338(97%) | 327/338(97%) | 327/338(97%) |
| *Diaporthe compacta* strain LC3078  (KP267924) | 324/338(96%) | 327/338(97%) | 327/338(97%) | 327/338(97%) |
| *Diaporthe compacta* isolate ZJUE0380  (OP265584) | 338/349(97%) | 341/349(98%) | 341/349(98%) | 341/349(98%) |
| *Diaporthe compacta* isolate ZJUE0403  (OP265585) | 338/349(97%) | 341/349(98%) | 341/349(98%) | 341/349(98%) |
| *Diaporthe sambucusii* strain CFCC 51986  (KY852507) | 331/340(97%) | 334/340(98%) | 334/340(98%) | 334/340(98%) |
| *Diaporthe sambucusii* strain CFCC51987  (KY852508) | 331/340(97%) | 334/340(98%) | 334/340(98%) | 334/340(98%) |
| *Diaporthe ganjae* strain CBS 180.91  (KC343838) | 313/331(95%) | 313/331(95%) | 313/331(95%) | 313/331(95%) |
| *Diaporthe manihotia* strain CBS 505.76  (KC343864) | 323/359(90%) | 326/359(91%) | 326/359(91%) | 326/359(91%) |
| *Diaporthe manihotia* culture MFLU:19-2826  (MW014359) | 325/358(91%) | 328/358(92%) | 328/358(92%) | 328/358(92%) |

^1^ 4 isolates and GenBank accession Numbers.

^2^ the reference strains and GenBank accession Numbers.

^3^ Identities.

**Table S8** Morphology data of *Diaporthe betae*

| isolate | Alpha conidia | | Gamma conidia | | Conidiophores | |
| --- | --- | --- | --- | --- | --- | --- |
| HHL19101101S2D1 | length(μm) | width(μm) | length(μm) | width(μm) | length(μm) | width(μm) |
|  | 9.04 | 2.53 | 9.47 | 2.46 | 18.14 | 3.42 |
|  | 8.69 | 2.75 | 8.14 | 3.1 | 17.94 | 3.32 |
|  | 8.62 | 2.83 | 14.5 | 3.7 | 16.4 | 3.23 |
|  | 7.33 | 2.93 | 9.76 | 3.45 | 16.38 | 3.2 |
|  | 7.6 | 3.45 | 9.91 | 3.35 | 16.14 | 3.19 |
|  | 7.57 | 3.42 | 10.66 | 2.98 | 15.96 | 3.11 |
|  | 7.6 | 2.93 | 9.8 | 3.11 | 15.8 | 3.11 |
|  | 7.54 | 3.18 | 8.91 | 3.42 | 15.77 | 3.06 |
|  | 8.11 | 3.59 | 9.14 | 2.77 | 15.66 | 3.03 |
|  | 8.04 | 2.93 | 10.01 | 3.05 | 15.42 | 2.99 |
|  | 7.87 | 3.33 | 9.8 | 3.4 | 15.37 | 2.99 |
|  | 7.75 | 3.73 | 11.39 | 2.95 | 15.17 | 2.98 |
|  | 7.86 | 3.5 | 9.27 | 3.11 | 14.94 | 2.88 |
|  | 7.17 | 3.12 | 10.49 | 2.78 | 14.86 | 2.85 |
|  | 6.53 | 3.15 | 9.92 | 3.22 | 14.84 | 2.81 |
|  | 7.6 | 2.76 | 10.18 | 2.97 | 14.78 | 2.81 |
|  | 7.99 | 3.35 | 10.83 | 2.97 | 14.57 | 2.79 |
|  | 6.8 | 3.24 | 9.79 | 3.22 | 14.42 | 2.73 |
|  | 10.15 | 3.98 | 11.96 | 3.17 | 14.17 | 2.68 |
|  | 8.29 | 3.67 | 14.95 | 3.11 | 14.12 | 2.59 |
|  | 8.38 | 2.81 | 11.32 | 3.35 | 14.11 | 2.53 |
|  | 7.85 | 3.97 | 11.22 | 3.28 | 14.07 | 2.5 |
|  | 8.57 | 3.4 | 12.97 | 3.27 | 13.92 | 2.5 |
|  | 7.69 | 3.17 | 13.65 | 2.95 | 13.7 | 2.49 |
|  | 8.23 | 3.02 | 13.48 | 1.94 | 13.56 | 2.47 |
|  | 7.16 | 3.22 | 10.07 | 2.73 | 13.4 | 2.35 |
|  | 7.88 | 3.09 | 10.02 | 3.06 | 13.28 | 2.34 |
|  | 7.87 | 3.01 | 9.69 | 3.02 | 13.01 | 2.24 |
|  | 7.58 | 3.06 | 10.09 | 3 | 12.93 | 2.23 |
|  | 7.49 | 3.18 | 10.18 | 3.02 | 12.72 | 2.23 |
|  | 7.09 | 2.59 |  |  | 12.6 | 2.07 |
|  | 7.98 | 2.77 |  |  | 12.2 | 2.05 |
|  | 6.84 | 3.05 |  |  | 11.89 | 1.95 |
|  | 8.25 | 3.27 |  |  | 11.86 | 1.93 |
|  | 8.14 | 3.18 |  |  | 11.48 | 1.89 |
|  | 8.25 | 3.32 |  |  | 11.39 | 1.88 |
|  | 8.95 | 4.02 |  |  | 10.77 | 1.84 |
|  | 9.08 | 3.39 |  |  | 10.56 | 1.8 |
|  | 8.14 | 3.33 |  |  | 10.29 | 1.79 |
|  | 7.72 | 3.47 |  |  | 10.18 | 1.77 |
|  | 7.64 | 3.01 |  |  | 9.98 | 1.76 |
|  | 8.03 | 3.01 |  |  | 9.87 | 1.68 |
|  | 8.28 | 3.25 |  |  | 9.66 | 1.62 |
|  | 7.96 | 3.09 |  |  | 9.2 | 1.54 |
|  | 8.23 | 3.32 |  |  | 8.95 | 1.52 |
|  | 8.23 | 3.49 |  |  | 8.24 | 1.52 |
|  | 8.12 | 3.63 |  |  | 8.19 | 1.49 |
|  | 7.37 | 3.35 |  |  | 7.88 | 1.37 |
|  | 8.05 | 3.42 |  |  | 7.56 | 1.25 |
|  | 8.27 | 3.22 |  |  | 6.92 | 0.9 |
